# Anti-malaria drug artesunate prevents development of amyloid-β pathology in mice by upregulating PICALM at the blood-brain barrier

**DOI:** 10.1186/s13024-023-00597-5

**Published:** 2023-01-27

**Authors:** Kassandra Kisler, Abhay P. Sagare, Divna Lazic, Sam Bazzi, Erica Lawson, Ching-Ju Hsu, Yaoming Wang, Anita Ramanathan, Amy R. Nelson, Zhen Zhao, Berislav V. Zlokovic

**Affiliations:** Department of Physiology and Neuroscience and the Zilkha Neurogenetic Institute, Keck School of Medicine of the University of Southern California, 1501 San Pablo St, Los Angeles, CA 90089 USA

**Keywords:** Artesunate, PICALM, Blood-brain barrier, Alzheimer’s disease, Amyloid-β, Mice

## Abstract

**Background:**

*PICALM* is one of the most significant susceptibility factors for Alzheimer’s disease (AD). In humans and mice, PICALM is highly expressed in brain endothelium. PICALM endothelial levels are reduced in AD brains. PICALM controls several steps in Aβ transcytosis across the blood-brain barrier (BBB). Its loss from brain endothelium in mice diminishes Aβ clearance at the BBB, which worsens Aβ pathology, but is reversible by endothelial PICALM re-expression. Thus, increasing PICALM at the BBB holds potential to slow down development of Aβ pathology.

**Methods:**

To identify a drug that could increase PICALM expression, we screened a library of 2007 FDA-approved drugs in HEK293t cells expressing luciferase driven by a human *PICALM* promoter, followed by a secondary mRNA screen in human Eahy926 endothelial cell line. In vivo studies with the lead hit were carried out in *Picalm*-deficient (*Picalm*^+/−^) mice, *Picalm*^+/−^; *5XFAD* mice and *Picalm*^lox/lox^; *Cdh5*-Cre; *5XFAD* mice with endothelial-specific *Picalm* knockout. We studied PICALM expression at the BBB, Aβ pathology and clearance from brain to blood, cerebral blood flow (CBF) responses, BBB integrity and behavior.

**Results:**

Our screen identified anti-malaria drug artesunate as the lead hit. Artesunate elevated PICALM mRNA and protein levels in Eahy926 endothelial cells and in vivo in brain capillaries of *Picalm*^+/−^ mice by 2–3-fold. Artesunate treatment (32 mg/kg/day for 2 months) of 3-month old *Picalm*^+/−^; *5XFAD* mice compared to vehicle increased brain capillary PICALM levels by 2-fold, and reduced Aβ42 and Aβ40 levels and Aβ and thioflavin S-load in the cortex and hippocampus, and vascular Aβ load by 34–51%. Artesunate also increased circulating Aβ42 and Aβ40 levels by 2-fold confirming accelerated Aβ clearance from brain to blood. Consistent with reduced Aβ pathology, treatment of *Picalm*^+/−^; *5XFAD* mice with artesunate improved CBF responses, BBB integrity and behavior on novel object location and recognition, burrowing and nesting. Endothelial-specific knockout of PICALM abolished all beneficial effects of artesunate in *5XFAD* mice indicating that endothelial PICALM is required for its therapeutic effects.

**Conclusions:**

Artesunate increases PICALM levels and Aβ clearance at the BBB which prevents development of Aβ pathology and functional deficits in mice and holds potential for translation to human AD.

**Supplementary Information:**

The online version contains supplementary material available at 10.1186/s13024-023-00597-5.

## Background

*PICALM*, the gene encoding phosphatidylinositol binding clathrin assembly protein PICALM [1, 2], is a highly validated genetic risk factor for late onset Alzheimer’s disease (LOAD) [3–23], and one of the most significant susceptibility factors for LOAD after *APOE* and *BIN1* [3, 4, 11, 15, 20]. PICALM regulates endocytosis and internalization of cell receptors [24–28], and intracellular trafficking of functionally different proteins [28–30]. In brain, PICALM is highly expressed in endothelial cells both in humans and mice [28, 31, 32], as well as in neurons [27, 33, 34] and microglia [33, 35]. Despite strong association with AD, the role of *PICALM* in disease pathogenesis remains elusive. We also do not have an effective PICALM-based therapy for AD.

PICALM may affect disease by different mechanisms. This includes modifying trafficking of amyloid-β (Aβ) precursor protein (APP) [3], protecting neurons by reversing Aβ effects on clathrin–mediated endocytosis [27], directing APP transport to autophagosomes [36], influencing Aβ42/Aβ40 ratio in neurons via clathrin-mediated endocytosis of γ-secretase [34], and/or controlling tau-mediated neurodegeneration [37] that spreads throughout neurons by low density lipoprotein receptor related protein 1 (LRP1)-mediated endocytosis [38]. In endothelium, PICALM regulates Aβ endocytosis by interacting with LRP1, a key Aβ clearance receptor at the blood-brain barrier (BBB) [28, 39–46], and by guiding Aβ trafficking post-internalization to small Rab GTPases Rab5 and Rab11, which results in Aβ trans-endothelial transcytosis and clearance across the BBB [28].

Previous work reported that PICALM endothelial levels are reduced by 60% in AD brains [28, 31, 32], which inversely correlated with Aβ load, Braak stage, clinical dementia rating score, and positively correlated with Mini Mental State Examination [28]. In AD-derived human BBB endothelial monolayers, reduced PICALM levels led to diminished Aβ clearance across the BBB which was reversible by adenoviral–mediated *PICALM* gene transfer. iPSC-derived human endothelial cells carrying the non-protective allele of the frequently studied single nucleotide polymorphism (SNP) *rs3851179 PICALM* variant [13, 14, 16, 17, 21, 22, 28] exhibited lower PICALM levels and reduced Aβ clearance across human endothelial BBB monolayers [28]. Moreover, *Picalm* deficiency in *APP*^sw/0^ overexpressing mice significantly diminished Aβ clearance across the murine BBB in vivo and accelerated Aβ pathology, in a manner that was reversible by endothelial PICALM re-expression [28]. All these findings support the concept that strategies to increase endothelial PICALM levels may increase Aβ clearance across the BBB and delay Aβ accumulation in brain.

In searching for a drug that could increase PICALM levels, we screened 2007 compounds from FDA-approved drug libraries because these drugs would likely be easier and faster to repurpose for a new therapeutic use than unapproved compounds. Our screen identified anti-malaria drug artesunate as the lead hit. Using *Picalm*-deficient *Picalm*^*+/−*^; *5XFAD* mice and 5XFAD mice with a complete PICALM endothelial-specific deletion, we found that artesunate treatment prevents Aβ accumulation in brain and improves functional outcome in 5XFAD mice by increasing PICALM levels in brain capillary endothelium and Aβ clearance at the BBB.

## Methods

### Drug screen and in vitro hit conformation

#### Generation of stable cell line

HEK293t cells were grown in DMEM supplemented with 10% FBS, 1 mM Na pyruvate, and 1% penicillin/streptomycin (all Thermo Fisher). Cells were transfected with a plasmid encoding both a secreted luciferase reporter driven by a human *PICALM* promotor and secreted alkaline phosphatase (SEAP) internal control driven by a CMV promotor (GeneCopoeia HPRM13888-PG04) using Lipofectamine 3000 (Thermo Fisher) following the manufacturer’s instructions. The plasmid also imparts puromycin resistance to the transfected cells. Transfected cells were maintained in media with puromycin (5 μg/mL; Sigma) and selected for monoclonal integration and expression of the *PICALM*/SEAP reporter following guidelines published by Lonza (http://www.lonza.com/go/literature/72). Expression was confirmed by luciferase assay as described below.

#### Drug screen using PICALM luciferase reporter

Cells were plated at a density of 5 × 10^4^ per well in 96 well plates and allowed to grow for 24 hours. Cell culture media was then replaced with media containing 3 μM of test drug. FDA-approved drugs used in the screen were sourced from the NIH Clinical Drug Collections NCC-202 and NCC-105 (727 compounds) and the MicroSource Spectrum Collection (1280 compounds) through the USC Choi Family Therapeutic Screening Facility. DMSO, negative and positive control wells were included for each plate. Media was collected 24 hours after drug treatment, aliquoted and stored at − 80 °C. Each drug was screened in triplicate. Luciferase and SEAP content were determined using a commercially available kit (GeneCopoeia Secrete-Pair Dual Luminescence Assay Kit; catalog #LF033) following the manufacturer’s instructions. Briefly, media samples were thawed to room temperature (RT). For each sample, 10 μL of media was added to a white opaque 96 well plate and 100 μL of luciferase substrate buffer added to the well. Plates were allowed to incubate at RT protected from light for 5 minutes, then read on a Victor3 (Perkin Elmer) plate reader. SEAP levels were determined similarly with a separate 10 μL media aliquot, heat inactivated at 65 °C for 15 min prior to addition of SEAP substrate buffer. SEAP assay plates were allowed to incubate for 15 min in SEAP substrate buffer before reading.

**Fig. 1:**
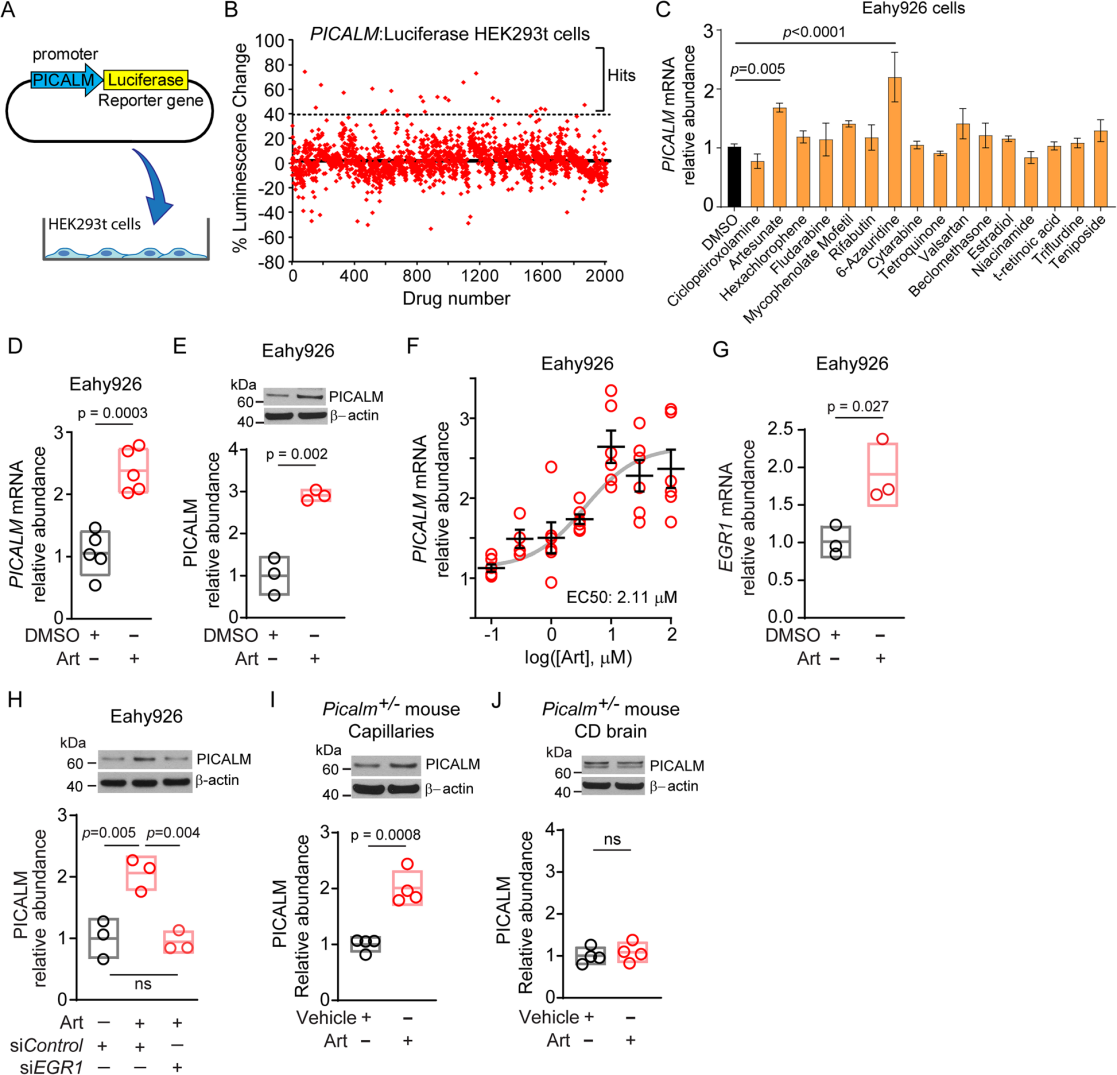
A screen of 2007 FDA-approved compounds revealed artesunate as a lead drug candidate upregulating PICALM in cell assays and mouse brain capillaries in vivo. **A** A *PICALM* promotor-driven secreted luciferase plasmid was integrated into HEK293t cells to create a stable luciferase reporter cell line for *PICALM* drug screening. **B** Using the HEK293t luciferase reporter line, 2007 FDA-approved drugs were evaluated for *PICALM* upregulation. Screen was run in triplicate at 3 μM drug concentration. Each point represents luciferase signal for each drug in the screen relative to SEAP internal control and normalized to DMSO control luciferase signal in the absence of drugs. Drugs with luciferase increases over 3 SD above the mean DMSO-normalized luciferase/SEAP value for all drugs (dashed line) were considered “hits” and further evaluated (see Methods and Results). **C** Secondary mRNA evaluation of drug hits by RT-qPCR in human Eahy926 endothelial cells. Relative abundance of *PICALM* mRNA after incubation of Eahy926 endothelial cells with top drug hits at 3 μM for 24 hours, normalized by the house-keeping gene glyceraldehyde 3-phosphate dehydrogenase (*GAPDH*) mRNA levels and compared to DMSO vehicle. Data are mean ± SEM; *n* = 3 independent cultures per condition. Significance by ANOVA vs DMSO followed by Dunnett’s multiple comparisons test. **D**, **E** Relative abundance of PICALM mRNA (**D**) and protein levels (**E**) after incubation of Eahy926 endothelial cells with top drug hit artesunate (Art) at 3 μM for 24 hours. The relative abundance of *PICALM* mRNA was normalized by the *GAPDH* mRNA levels (**D**) or β-actin protein (**E**), and compared to DMSO vehicle. *n* = 5 replicates for **D**, and *n* = 3 replicates for **E**. **F** Relative abundance of *PICALM* mRNA normalized by the *GAPDH* levels in the presence of different concentrations of artesunate. Bars represent mean ± SEM at each concentration studied; *n* = 4–6 for each concentration. Individual values indicated by circles; gray line shows a sigmoid curve fit to the data for determination of the EC50 value. **G** Relative abundance of *EGR1* mRNA after incubation of Eahy926 endothelial cells with 3 μM artesunate for 24 hours. The relative abundance of *EGR1* mRNA was normalized by *GAPDH* mRNA levels, and compared to DMSO vehicle. n = 3 replicates. **H** Relative abundance of PICALM protein levels after incubation of Eahy926 endothelial cells with 3 μM Art for 24 hours after silencing *EGR1* (si*EGR1*) or in the presence of control siRNA (si*Control*). n = 3 replicates. **I-J** PICALM protein levels in brain capillaries (**I**) and capillary-depleted brains (**J**) from *Picalm*^*+/−*^ mice treated i.p. with 32 mg/kg artesunate or vehicle for 1 week. n = 4 mice per group. Individual replicates in **D**, **E**, **G**, **H,** or mice in **I**, **J** indicated by circles; All data in these panels are mean ± SD. Significance in **D**, **E**, **G**, **I** and **J** by Student’s two-tail t-test. Significance in **H**, by one-way ANOVA followed by Tukey post-hoc test. Full blots for E, H-J shown in supp. Fig. 5

SEAP-normalized luciferase (luciferase/SEAP) signal was calculated for each replicate and then averaged across replicates for each drug. This average was then normalized to DMSO-treated control in the absence of any drug (averaged value from 4 DMSO-treated wells per 96-well plate). The mean DMSO-treated control luciferase/SEAP level in the absence of any drug was set as 1, and luciferase/SEAP DMSO-normalized values were averaged across all studied drugs (2007 total). SD and mean + 3SD across all drugs were calculated. A drug was considered a “hit” if its DMSO-normalized luciferase/SEAP value was greater than 3 SD above the mean DMSO-normalized luciferase/SEAP value for all drugs in the screen, which is a commonly used cut-off in drug screen hit evaluations [47–49]. Data are presented in Fig. 1B and Fig. S1A as percent luminescence change relative to DMSO control levels.

#### Real-time quantitative polymerase chain reaction (RT-qPCR)

For in vitro studies, relative messenger ribonucleic acid (mRNA) abundance of human *PICALM* after treatment with drug screen hits, or early growth response 1 (*EGR1*) after artesunate treatment and/or *EGR1* silencing (see below), were determined by RT-qPCR in Eahy926 human endothelial cell line (ATCC CRL-2922). Cells were plated in 12-well plates and grown to ~ 90% confluence. Drugs were added at 3 μM concentration and incubated for 24 hours. DMSO treated cells were used as control. Cells were collected from each well and total RNA was prepared using RNeasy kit (Qiagen, 74104) and real-time qPCR amplification was performed using one-step SYBR green qPCR kit (QuantaBio, 95087). Relative abundance was calculated using ΔΔCt method normalized to the house-keeping gene glyceraldehyde 3-phosphate dehydrogenase (*Gapdh*) as we described [50, 51]. Results were then normalized to DMSO levels. Dose-response assays were performed similarly by treating Eahy926 cells with varying doses of artesunate. The following primers were used:

**Table:** 

Gene	Forward primer	Reverse primer
*PICALM*	5′- CTCCTGTATCCACCTCAGCA-3’	5′- CTGCTGCAAATCAAGCAGAT-3’
*EGR1*	5′- CCACGCCGAACACTGACATT-3’	5′- GAGGGGTTAGCGAAGGCTG-3’
*GAPDH*	5′- ACCACAGTCCATGCCATCAC-3′	5′- TCCACCACCCTGTTGCTGTA-3′

#### EGR1 silencing

Eahy926 human endothelial cells were plated as above, and transfected with control or *EGR1* siRNA (D-001910-10-50 and E-006526-00-0010, respectively, Dharmacon/Horizon Discovery), similar to previously described [52]. For in vitro studies, 48 hours after silencing, the cells were treated with artesunate as above for 24 hours.

### Animals

**Fig. 2:**
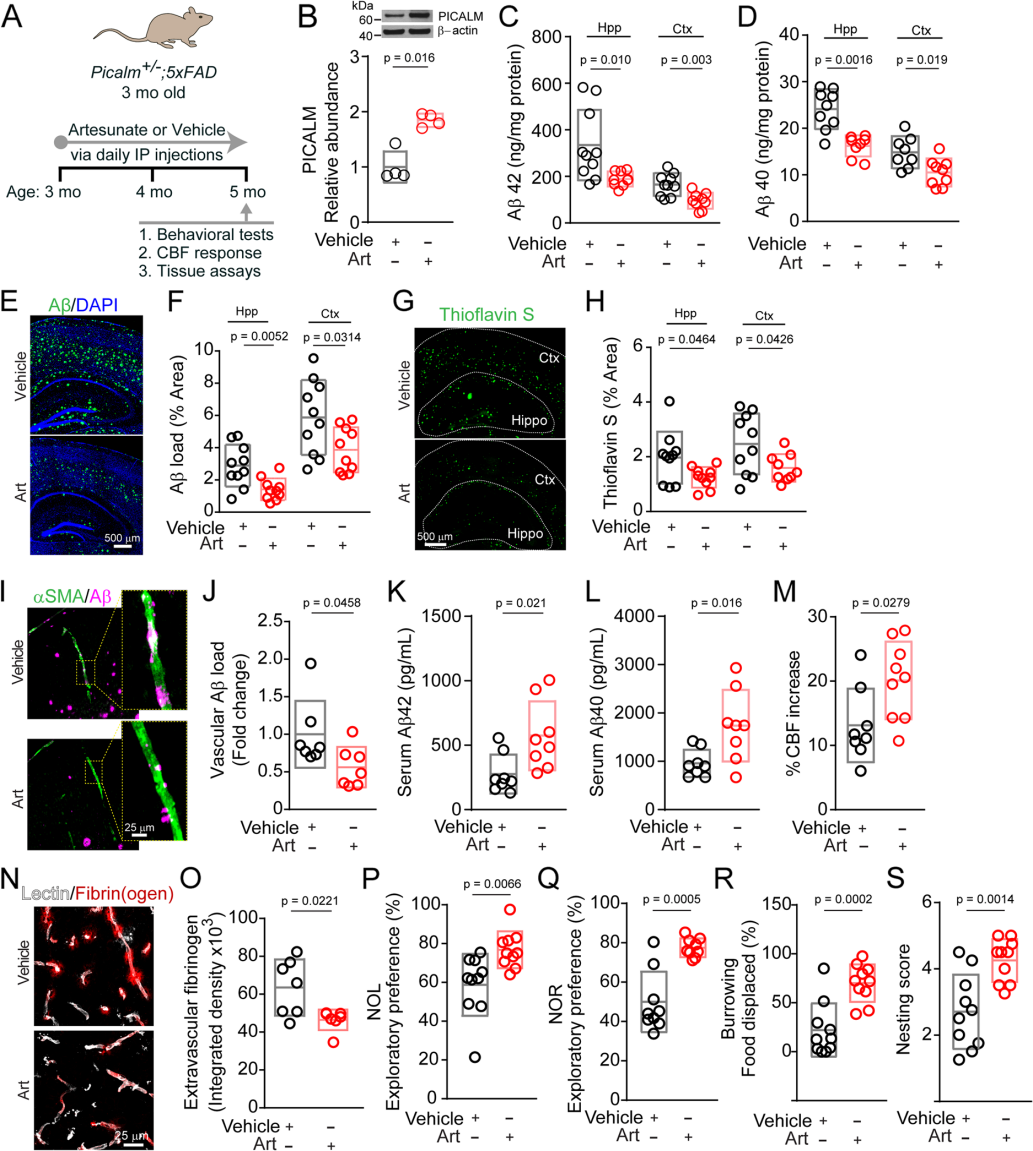
Artesunate increases PICALM levels in brain capillaries, slows development of Aβ pathology, increases brain-to-blood clearance of Aβ, and improves functional outcome in *Picalm*^+/−^; *5XFAD* mice. **A** Experimental treatment paradigm for *Picalm*^+/−^; *5XFAD* mice with artesunate (Art) or vehicle. Mice were injected i.p. with 32 mg/kg/day Art or vehicle for 2 months, starting at 3 months of age, followed by behavior tests, evaluation of cerebral blood flow (CBF) responses, and tissue collection and assays at 5 months of age. **B** PICALM protein relative expression levels in brain capillaries isolated from *Picalm*^+/−^; *5XFAD* mice treated with vehicle or Art. n = 4 mice per condition. **C**, **D** Amyloid-β 42 (Aβ42) (**C**) and Aβ40 (**D**) levels in the hippocampus (Hpp) and cortex (Ctx) in *Picalm*^+/−^; *5XFAD* mice treated with vehicle or Art. *n* = 8–10 mice per condition (**C**: Hpp and Ctx Vehicle: 5 male, 5 female; Hpp and Ctx Art: 5 male, 4 female. **D**: Hpp Vehicle: 4 male, 5 male; Hpp Art: 4 male, 4 female; Ctx Vehicle: 4 male, 4 female; Ctx Art: 5 male, 4 female). **E**, **F** Representative images of Aβ immunostaining (**E**), and quantification of Aβ load in the Hpp and Ctx (**F**) in *Picalm*^+/−^; *5XFAD* mice treated with vehicle or Art. Scale bar in (**E**) is 500 μm. *n* = 10 mice per condition (Hpp and Ctx Vehicle: 5 male, 5 female; Hpp and Ctx Art: 6 male, 4 female). **G**, **H** Representative images of Thioflavin S-positive amyloid deposits (**G**) and quantification of Thioflavin S amyloid plaque load in the Hpp and Ctx (**H**) in *Picalm*^+/−^; *5XFAD* mice treated with vehicle or Art. Scale bar in (**G**) is 500 μm. *n* = 9–10 mice per condition (Hpp and Ctx Vehicle: 5 male, 5 female; Hpp Art: 6 male, 4 female; Ctx Art: 6 male, 3 female). **I**, **J** Representative images (**I**) and quantification (**J**) of Aβ vascular load in small pial arteries and penetrating cortical arterioles in *Picalm*^+/−^; *5XFAD* mice treated with vehicle or Art. Aβ vascular load is expressed as a fraction of Aβ-positive area relative to α-smooth muscle actin (αSMA)-positive area of the vessel wall, normalized to the vehicle-treated group. Scale bar in (**I**) is 25 μm. *n* = 7 mice per condition. **K**, **L** Serum Aβ42 (**K**) and Aβ40 (**L**) levels in *Picalm*^+/−^; *5XFAD* mice treated with vehicle or Art. n = 8 mice per condition (4 male, 4 female). **M** Cerebral blood flow (CBF) response to whisker stimulation in in *Picalm*^+/−^; *5XFAD* mice treated with vehicle or Art. n = 8–9 mice per condition (Vehicle: 4 male, 4 female; Art: 6 male, 3 female). **N**, **O** Representative images (**N**) and quantification (**O**) of blood-derived fibrin/fibrinogen extravascular leakage in the brain parenchyma in *Picalm*^+/−^; *5XFAD* mice treated with vehicle or Art. Scale bar in (**N**) is 25 μm. n = 7 mice per condition. **P**-**S** Novel object location (**P**) and recognition (**Q**), burrowing (**R**), and nesting score (**S**) in *Picalm*^+/−^; *5XFAD* mice treated with vehicle or Art. n = 9–10 mice per condition (**P**, **R**, and **S**: Vehicle: 5 male, 5 female and Art: 6 male, 4 female; **Q**: Vehicle: 4 male, 5 female and Art: 6 male, 3 female). Single points per mouse indicated by circles in **B-D**, **F**, **H, J-M**, **O**, **P-S,** with boxes representing mean ± SD. Significance determined by Student’s two-tail t-test in panels **B**, **F**, **H**, **K-M**, **R**, and **S**, or Mann-Whitney U test in panels **C, D**, **K**, **O-Q**. Full blots for B shown in supp. Fig. 5

**Fig. 3:**
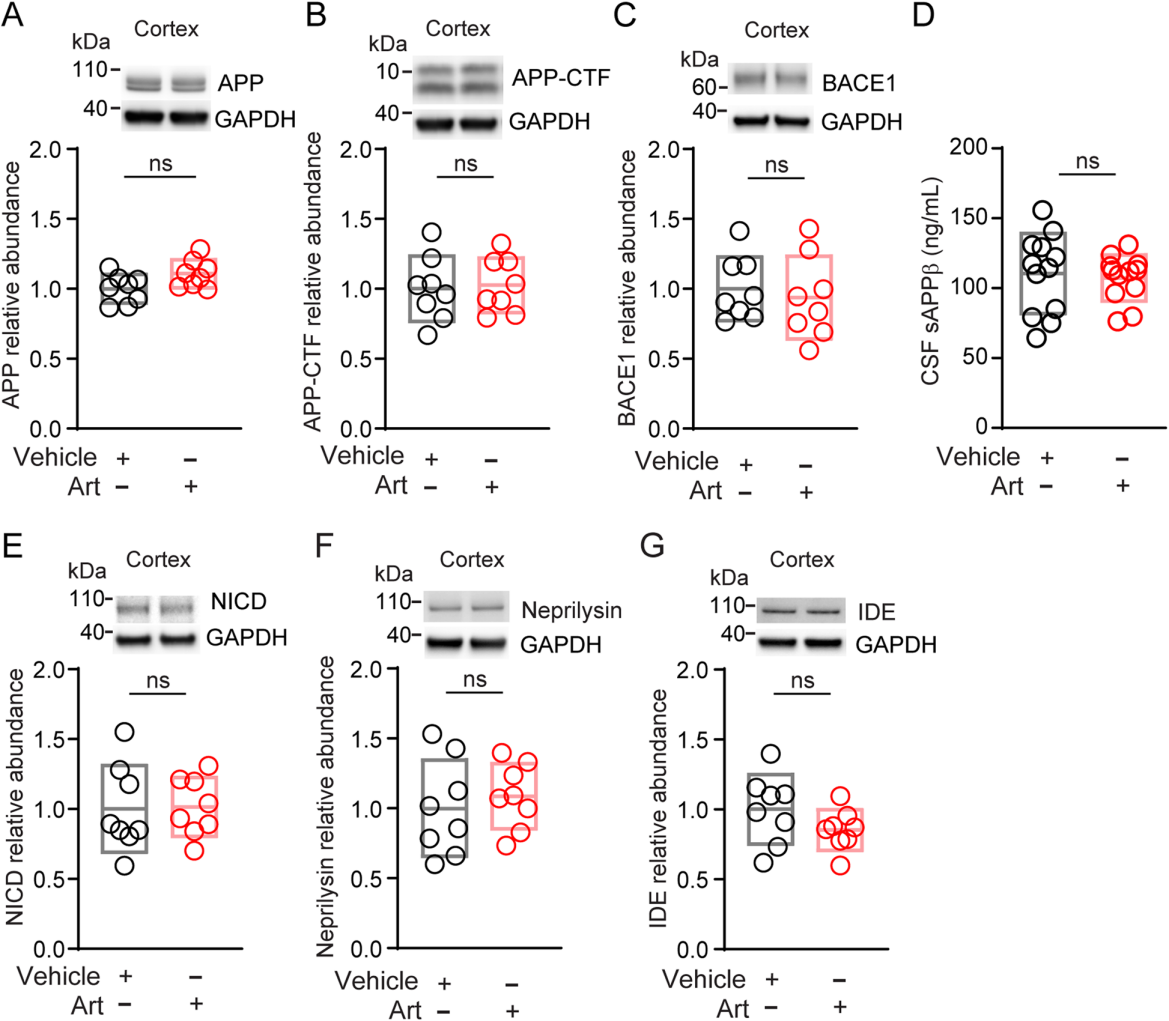
Artesunate does not alter the levels of Aβ processing proteins and Aβ clearance enzymes in *Picalm*^+/−^; *5XFAD* mice. **A** amyloid precursor protein (APP) abundance in cortex, **B** APP C-terminal fragment (APP-CTF) abundance in cortex. **C** β-secretase (BACE1) abundance in cortex, **D** soluble APP-β (sAPPβ) levels in cerebrospinal fluid (CSF), **E** γ–secretase activity as determined by the production of Notch intracellular domain (NICD) fragment from Notch protein, indicated by NICD abundance in cortex, **F** neprilysin abundance in cortex, and **G** insulin degrading enzyme (IDE) abundance in cortex of *Picalm*^+/−^; *5X FAD* mice treated with artesunate or vehicle as in Fig. 2A. The relative abundance of proteins was normalized by the house-keeping gene glyceraldehyde 3-phosphate dehydrogenase (GAPDH) protein. Single points per mouse indicated by circles, with boxes representing mean ± SD. **A-E**, **G** n = 8 per group; **F** n = 7–8 per group. ns = non-significant by two-tailed t-test. Full blots for A-C, E-G shown in supp. Fig. 5

**Fig. 4:**
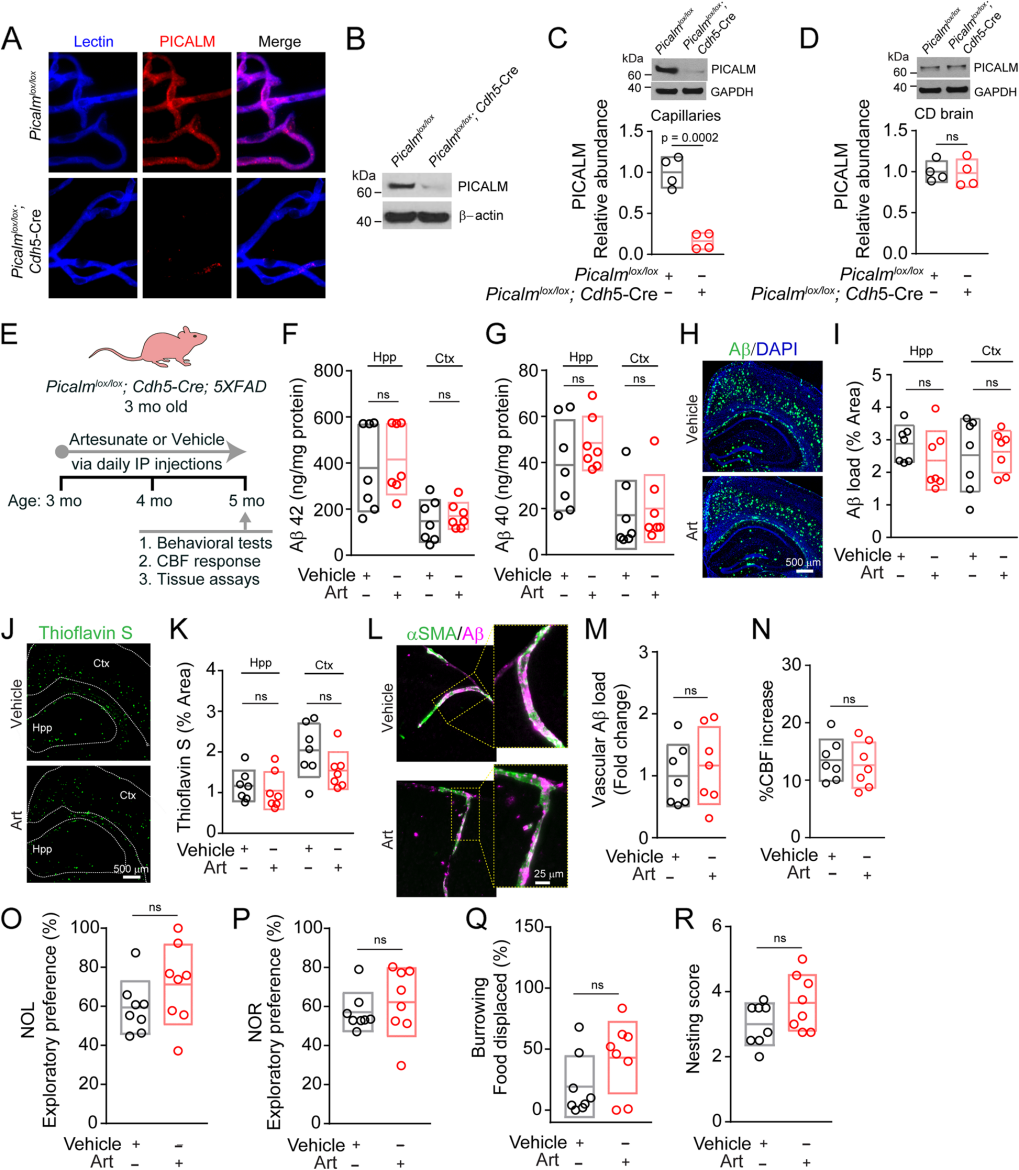
Complete endothelial knockout of PICALM eliminates all beneficial effects of artesunate in *5XFAD* mice. **A** PICALM immunostaining is barely detectable in lectin-positive endothelium in isolated brain capillaries from *Picalm*^lox/lox^; *Cdh5*-Cre mice compared to *Picalm*^lox/lox^ littermate controls. Immunostaining is representative of n = 3 for each mouse line. **B** PICALM immunoblotting of endothelial cells isolated from brain capillaries from *Picalm*^lox/lox^; *Cdh5*-Cre and *Picalm*^lox/lox^ mice. The blot is representative of *n* = 2 cultures for each mouse line. **C**, **D** PICALM immunoblotting of brain capillaries (**C**) and capillary depleted (CD) brain (**D**) isolated from *Picalm*^lox/lox^; *Cdh5*-Cre and *Picalm*^lox/lox^ mice. n = 4 for each mouse line. **E** Experimental treatment paradigm for *Picalm*^lox/lox^; *Cdh5*-Cre; *5XFAD* mice with artesunate (Art) or vehicle. Mice were injected i.p. with 32 mg/kg/day Art or vehicle for 2 months, starting at 3 months of age, followed by behavior tests, evaluation of cerebral blood flow (CBF) responses, and tissue collection and assays at 5 mo of age. **F**, **G** Amyloid-β 42 (Aβ 42) (**F**) and Aβ 40 (**G**) levels in the hippocampus (Hpp) and cortex (Ctx) in *Picalm*^lox/lox^; *Cdh5*-Cre; *5XFAD* mice treated with vehicle or artesunate (Art). **H**, **I** Representative images of Aβ immunostaining (**H**), and quantification of Aβ load in the Hpp and Ctx (**I**) in *Picalm*^lox/lox^; *Cdh5*-Cre; *5XFAD* mice treated with vehicle or Art. Scale bar in (**H**) is 500 μm. **J**, **K** Representative images of Thioflavin S-positive amyloid deposits (**J**) and quantification of Thioflavin S amyloid load in the Hpp and Ctx (**K**) in *Picalm*^lox/lox^; *Cdh5*-Cre; *5XFAD* mice treated with vehicle or Art. Scale bar in (**I**) is 500 μm. **L**, **M** Representative images (**L**) and quantification (**M**) of Aβ vascular load in small pial arteries and penetrating cortical arterioles in *Picalm*^lox/lox^; *Cdh5*-Cre; *5XFAD* mice treated with vehicle or Art. Aβ vascular load is expressed as fraction of Aβ-positive area relative to α-smooth muscle actin (αSMA)-positive area of the vessel wall, normalized to the vehicle-treated group. Scale bar in (**L**) is 25 μm. (**N**) Cerebral blood flow (CBF) response to whisker stimulation in *Picalm*^lox/lox^; *Cdh5*-Cre; *5XFAD* mice treated with vehicle or Art. **O-R** Novel object location (**O**) and recognition (**P**), burrowing (**Q**), and nesting score (**R**) in *Picalm*^lox/lox^; *Cdh5*-Cre; *5XFAD* mice treated with vehicle or Art. In **F**, **G**, **I**, **K**, **M**, **N**, n = 7 mice per condition (Vehicle: 4 male, 3 female; Art: 5 male, 2 female). In **O-R**, *n* = 8 mice per condition (Vehicle: 4 male, 4 female; Art: 6 male, 2 female). Single points per mouse indicated by circles in **C**, **D**, **F**, **G**, **I**, **K**, **M-R**, with boxes representing mean ± SD. Significance determined by two-tail t-test. Full blots for B-D shown in supp. Fig. 5

For mouse data shown in Fig. 1I and J, 3-4 mo old *Picalm*^*+/−*^ mice lacking one copy of *Picalm* gene [28, 53] were used. Transgenic mice with five familial Alzheimer’s disease (AD) mutations (5XFAD) used in this study were purchased from The Jackson Laboratory (Cat. No 34840). 5XFAD mice carry K670N/M67L (Swedish), I716V (Florida), and V717I (London) mutations in human APP (695), and M146L and L286V mutations in human PSEN1 gene in the brain [54]. Both transgenes are regulated by neuronal mouse Thy1 promoter and express transgenes exclusively in neurons [54]. Our preliminary data indicated that sex did not influence the development of Aβ pathology in 5XFAD mice at a disease stage that we studied consistent with some previous reports in 5XFAD mice [54–57]. Therefore, both female and male mice were used in the study. 5XFAD mice were crossed to *Picalm*^*+/−*^ mice to generate PICALM-deficient 5XFAD mice (*Picalm*^*+/−*^*;5XFAD*). Data generated using these mice appear in Figs. 2, 3, S2, S3, and S4. Untreated *5XFAD* littermates (5 mo old) were used for amyloid load characterization and comparisons shown in Fig. S2. Mice with an endothelial-specific PICALM deletion, *Picalm*^*lox/lox*^*; Cdh5-*Cre, were generated in our lab by crossing *Picalm*^*lox/lox*^ mice [53] with *Cdh5*-Cre mice (Jackson Laboratories stock #006137), and then crossed to 5XFAD mice to produce *Picalm*^*lox/lox*^*; Cdh5-*Cre; *5XFAD* mice. Data generated using these mice appear in Fig. 4. The animals were housed in plastic cages on a 12 h light cycle with ad libitum access to water and a standard laboratory diet. All procedures were approved by the Institutional Animal Care and Use Committee at the University of Southern California following National Institutes of Health guidelines.

### Artesunate treatment

Artesunate (Sigma) was prepared in isotonic saline with 5% NaHCO_3_ and administered to animals in a dosage of 32 mg/kg/day [58]. Artesunate solution or vehicle (saline with 5% NaHCO_3_) was administered daily by intraperitoneal injections in *Picalm*^*+/−*^ mice for 7 days for preliminary drug evaluation (Fig. 1I, J), or 3-month-old *Picalm*^*+/−*^*;5XFAD* mice and *Picalm*^*lox/lox*^*;Cdh5*-Cre; *5XFAD* mice for 2 months. Mice were weighed at the start of treatments, and weight monitored weekly over the duration of the treatments.

### Behavior

Before cerebral blood flow (CBF) studies and tissue collection, mice were studied for behavioral changes using novel object location, recognition, nesting, and burrowing tests that were performed as previously reported [50, 59, 60] with modifications described below.

#### Novel object location and recognition

Animals were habituated in a 30 × 30 × 30 cm arena for 30 minutes on two consecutive days. On the third day, after 10 minutes of habituation, animals were placed in the arena with 2 approximately 5 × 5 cm objects (blocks of similar size and shape) placed near the top left and right corners of the testing area. Animals were allowed to explore the objects and area within the arena for 10 minutes (training) then returned to their home cages. For novel object location (NOL), 1.5 hours after the training, one of the objects was relocated diagonally (novel) and the animals were reintroduced to the arena and allowed to explore the area for 3 minutes. For novel object recognition, 1.5 h after the completion of NOL, one of the objects was replaced with a new object (different shape and color) placed in the same location and the animals were allowed to explore the area within the arena for 5 min.

After each trial, the testing arena and the objects were thoroughly cleaned with 70% ethanol solution. All the trials, including habituation, were recorded with a high resolution camera and the amount of time each animal spent exploring the objects was analyzed and presented as % time spent with novel object/location over the sum of time spent with novel and old objects. Object exploration was defined as sniffing or touching an object with the snout at a critical distance of < 1 cm from object, as previously reported [45, 50]. Any animal that showed a preference for either of the two objects before replacement with the novel object/location, or explored both objects for less than 5 s was eliminated from the analysis.

#### Burrowing

To assess burrowing behavior, mice were individually placed in cages equipped with a polyvinyl chloride pipe burrow [60]. The burrow was filled with 200 g of mouse food pellets, and the mice were allowed to burrow for 2 h. The weight of the remaining food pellets inside the burrow was determined to obtain a measurement of the amount burrowed.

#### Nesting

To assess nest construction behavior, mice were individually placed in their home cages with a nestlet ~ 1 h before the dark phase. The nests were assessed the next morning and given a score of 1–5 based on their ability to shred the nestlet and build a nest [60].

### Cerebral blood flow studies

Before tissue collection and after behavioral studies, mice were anesthetized (~ 1% isoflurane) and cerebral blood flow (CBF) responses to vibrissal stimulation were determined using laser doppler flowmetry as described previously [50, 61]. CBF was recorded during stimulation and the percentage CBF increase was obtained by subtracting the baseline from the maximum CBF value reached during stimulus. A total of three trials were averaged for each mouse with 10 minute recovery periods between trials.

### Tissue collection

For tissue collection, mice were anesthetized intraperitoneally with 100 mg/kg ketamine and 10 mg/kg xylazine and transcardially perfused with ice-cold 0.01 M phosphate buffer saline (PBS), pH 7.4, containing 5 mM ethylenediaminetetraacetic acid (EDTA). Brains were rapidly removed, a portion of the frontal cortex was cut away for microvessel isolation. One hemisphere of the remaining tissue was embedded and frozen in optimal cutting temperature compound (OCT, Tissue-Tek), and the other hemisphere was snap frozen and saved for protein analysis. Microvessels were isolated and capillary depleted brain collected from the frontal cortex as previously described [62] and used for protein analyses.

For *Picalm*^*lox/lox*^*; Cdh5-*Cre characterization, microvessels were adhered to glass histology slides using a Cytospin III Cytocentrifuge (Shandon, Pittsburgh, PA, USA) and fixed in 4% PFA for 10 minutes for immunofluorescent analysis, or microvessel homogenates were immunoblotted for PICALM.

For *Picalm*^*lox/lox*^*; Cdh5-*Cre mouse endothelial cells, microvessels were digested (Collagenase/dispase, Roche 10,269,638,001), plated on gelatin-coated dishes and cultured in endothelial cell media (CellBiologics H1186) as previously described [63].

### Immunohistochemistry (IHC)

Frozen brain hemispheres from transcardially perfused mice were serially sectioned in the coronal plane on a cryostat (20 μm; Leica) and post-fixed with 4% paraformaldehyde (PFA) for 10 minutes. After washing with PBS, the sections were blocked in 5% normal donkey serum (Vector Laboratories)/0.3% Triton-X/0.01 M PBS for 1 hour and incubated with primary antibodies diluted in blocking solution overnight at 4 °C. We used the following antibodies: rabbit anti-human amyloid-β (Aβ) to detect Aβ deposits; mouse FITC conjugated α-smooth muscle actin (SMA) to visualize vascular smooth muscle cells and rabbit polyclonal anti-human fibrinogen that cross-reacts with mouse fibrionogen [51] (recognizes both monomeric form of fibrinogen as well as fibrinogen-derived fibrin polymers) to detect extravascular fibrinogen deposits. To visualize brain vessels, sections were incubated with Dylight 488-conjugated *Lycopersicon esculentum* lectin together with primary antibodies. After incubation in primary antibodies, sections were incubated with fluorophore-conjugated secondary antibodies (see Table 1 for details on antibodies used). Microvessel cytospin slides were prepared similarly. For detection of Aβ plaques, after PFA fixation the sections were incubated in 1% aqueous thioflavin-S (Sigma, T1892) for 5 minutes and rinsed in 80% ethanol, 95% ethanol and distilled water. All the slides were mounted with DAPI Fluoromount (Southern Biotech, 0100–20).

**Table 1:** Primary and secondary antibodies used for IHC

Primary antibody or lectin (manufacturer, catalog #, dilution used)	Secondary antibody (manufacturer, catalog #, dilution used)
*Human amyloid-β*	
Rabbit anti-human β-amyloid (Cell Signaling, 8243S, 1:500)	Alexa fluor 647-conjugated donkey anti-rabbit (Invitrogen, A-31573,1:500)
*Smooth muscle cells*	
Mouse FITC conjugated α-smooth muscle actin (SMA) (Sigma, clone 1A4, F3777, 1:500)	N/A
*Vasculature*	
Dylight 488-conjugated *L. esculentum* lectin (Vector Labs, DL-1174,1:200)	N/A
*Fibrinogen*	
Rabbit anti-human fibrinogen, cross-reacts with mouse fibrinogen [48] (Dako, A0080,1:400)	Alexa fluor 568-conjugated donkey anti-rabbit (Invitrogen, A-10042,1:500)
*PICALM*	
Goat anti-human PICALM, cross-reacts with mouse PICALM (Santa Cruz, sc-6433, 1:200)	Alexa fluor 568-conjugated donkey anti-goat (Invitrogen, A-11057,1:500)
*Microglia*	
Rabbit anti-mouse Iba1 (Wako, 019-19741; 1:500)	Alexa Fluor 568–conjugated donkey anti-rabbit (Invitrogen, A-10042, 1:500)
*Astrocytes*	
Rabbit anti-bovine GFAP, cross-reacts with mouse GFAP (Dako, Z0334, 1:500)	Alexa Fluor 568–conjugated donkey anti-rabbit (Invitrogen, A-10042, 1:500)

### Amyloid-β and thioflavin-S deposits

Amyloid-β-positive and thioflavin-S-positive area were determined using ImageJ software (US National Institutes of Health) as previously reported [50]. Images were taken on BZ9000 fluorescent microscope (Keyence) in single plain on 20x, subjected to threshold processing (Otsu) using ImageJ and the area % occupied by the signal in the image area was measured. In each animal, 5 randomly selected fields from the cortex and hippocampus were imaged and analyzed in 4 nonadjacent sections (∼100 μm apart).

### Vascular amyloid load (cerebral amyloid angiopathy, CAA) in small pial arteries and penetrating cortical arterioles

To visualize CAA, brain sections were double stained with α-smooth muscle actin (αSMA) and Aβ antibodies, and imaged on BZ9000 fluorescent microscope in single plain at 10x magnification, following previously described protocol [50, 64, 65]. In every image, percent area occupied with Aβ was divided by percent area occupied with αSMA to obtain percent Aβ vascular load reflecting development of CAA. Total of 10–15 vessels per animal in 4 sections and 5 images per section were analyzed.

### Extravascular fibrinogen deposits

Quantification was performed from maximum projections of 10 μm thick Z-stack images taken on BZ9000 fluorescent microscope subjected to threshold processing (Otsu) using ImageJ, and the amount of fibrinogen was determined as integrated density of the deposits on the abluminal side of the lectin-positive vessels, as described previously [50, 51]. Representative images were taken on a Nikon A1R confocal microscope with all imaging conditions kept identical for both groups. In each animal, 5 randomly selected fields from the cortex and hippocampus were analyzed in 4 nonadjacent sections (∼100 μm apart).

### Protein analyses

#### Aβ40 and Aβ42 specific enzyme-linked immunosorbent assay (ELISA)

Hippocampi and cortices were homogenized in ice-cold guanidine buffer (5 M guanidine hydrochloride/50 mM Tris HCl, pH 8), as described previously [66]. Human Aβ40 and Aβ42 levels in brain homogenates were determined using a Meso Scale Discovery assay (MSD, K15200E-1) following the manufacturer’s instructions, as previously reported [50]. Aβ40 and Aβ42 levels in serum were determined using the Meso Scale Discovery assay kit (MSD, K15199G), following the manufacturer’s instructions.

#### Soluble APPβ (sAPPβ) assay

Cerebrospinal fluid (CSF) collected from cisterna magna of anesthetized 5XFAD animal crosses was used to determine the levels of sAPPβ released from the Swedish variant of amyloid precursor protein (APP) by MSD assay (MSD, K151BUE-1), as previously reported [50].

#### Western blot

Eahy926 cells, isolated cortical microvessels or cortical brain tissue was resuspended in 20x RIPA buffer (50 mM Tris pH 8.0, 150 mM NaCl, 1% NP40, 0.1% SDS, 0.5% sodium deoxycholate and Roche protease inhibitor cocktail) and sonicated. After sonication the samples were centrifuged at 20,000 x g for 30 minutes, and supernatants were used for protein quantification (Thermo Fisher, 23,228). Samples were prepared with lithium dodecyl sulfate sample buffer (Invitrogen) and proteins (5–10 μg total protein loaded per sample) were separated by electrophoresis on NuPAGE Novex Bis-Tris precast 4–12% gradient gels (Thermo Fisher). After electrophoretic transfer, nitrocellulose membranes were blocked with blocking buffer (Thermo Fisher, 37,536) and incubated overnight at 4 °C with primary antibodies diluted in blocking solution. After washing with tris buffered saline containing 0.1% Tween 20 (TBST) membranes were incubated with horseradish peroxidase (HRP)-conjugated donkey anti-rabbit or anti-goat secondary antibody for 1 hour at room temperature (see Table 2 for details on antibodies used), washed again in TBST and treated for 5 minutes with Super Signal West Pico chemiluminescent substrate (Thermo Fisher) or Pierce ECL Western blotting chemiluminescent substrate (Thermo Fisher, 32,106). Membranes were either exposed to CL-XPosure film (Thermo Fisher) within the linear dynamic range of the film and developed in X-OMAT 3000 RA film processer (Kodak) and intensity of blots determined using ImageJ for quantification, or using a Carestream (KODAK) IS4000MM Pro Image Station digital chemiluminescence gel documentation instrument and accompanying Carestream Molecular Imaging Software. V 5.0 Software for quantification. The intensity of protein bands was normalized with respective loading control bands, as previously described [50]. Full blots for all blots presented in the figures are included in Fig. S5.

**Table 2:** Primary and secondary antibodies used for Immunoblotting

Primary antibody (manufacturer, catalog #, dilution used)	Secondary antibody (manufacturer, catalog #, dilution used)
*PICALM*	
Rabbit anti-human PICALM, cross-reacts with mouse PICALM (Sigma, HPA019061, 1:1000)	HRP-conjugated donkey anti-rabbit (Invitrogen, A16023,1:3000)
*β-secretase 1 (BACE1)*	
Rabbit anti-human BACE1, cross-reacts with mouse BACE1 (Cell Signaling, 5606, 1:1000)	HRP-conjugated donkey anti-rabbit (Invitrogen, A16023,1:3000)
*Amyloid precursor protein (APP) and APP C-terminal fragments (APP-CTFs)*	
Rabbit anti-human carboxy terminus of APP, cross-reacts with mouse APP and APP-CTFs (Cell Signaling, 76600S, 1:1000)	HRP-conjugated donkey anti-rabbit (Invitrogen, A16023,1:3000)
*Neprilysin*	
Goat anti-mouse neprilysin (R&D systems, AF1126, 1:1000)	HRP-conjugated donkey anti-goat (Invitrogen, A16005, 1:3000)
*Notch 1, Notch Intracellular Domain (NICD)*	
Rabbit anti-human N-terminal sequence of the cleaved NICD, cross-reacts with mouse NICD (Millipore Sigma, 07-1232, 1:1000)	HRP-conjugated donkey anti-rabbit (Invitrogen, A16023,1:3000)
*Insulin degrading enzyme (IDE)*	
Rabbit anti-rat insulin degrading enzyme (IDE), cross-reacts with mouse IDE (Millipore Sigma, PC730, 1:1000)	HRP-conjugated donkey anti-rabbit (Invitrogen, A16023,1:3000)
*Loading controls*	
Rabbit anti-human β-actin, cross-reacts with mouse β-actin (Cell Signaling, 4970S, 1:2000)	HRP-conjugated donkey anti-rabbit (Invitrogen, A16023,1:3000)
Rabbit anti-human GAPDH, cross-reacts with mouse GAPDH (Cell Signaling, 2118, 1:2000)	HRP-conjugated donkey anti-rabbit (Invitrogen, A16023,1:3000)

### Analysis of systemic biochemical parameters

For analysis of liver and kidney function ~ 200 μl of serum was collected from the heart and sent to IDEXX BioResearch, Sacramento, CA for screening (test code 6006).

### Statistical analysis

Sample sizes were calculated using nQUERY assuming a two-sided alpha level of 0.05, 80% power, and homogenous variances for the 2 samples to be compared, with the means and common standard deviation for different parameters predicted from published and our previous studies. GraphPad Prism 7.0 or later was used for statistical analysis calculations. F test was conducted to determine similarity in the variances between groups compared, and Kolmogorov-Smirnov normality test was used to test normality of the data sets. Statistical significance was analyzed by Student’s t-test or Mann-Whitney U test, as appropriate. For multiple comparisons, one-way analyses of variance (ANOVA) followed by Dunnett’s or Tukey multiple comparison test was performed, or two-way ANOVA followed by Bonferroni post-test as indicated in the figure legends. For all analyses, A *p* value of less than 0.05 was considered to be significant. Data are shown as scatter plots with single points per mouse and/or culture, and boxes representing mean ± SD, or bars graphs representing mean ± SEM or mean ± SD, as indicated in the figure legends.

## Results

### A screen of FDA-approved drugs reveals artesunate as a lead hit upregulating *PICALM*

To screen for drugs that upregulate *PICALM*, we first generated an HEK293t cell line stably expressing secreted luciferase, driven by a human *PICALM* promoter, and SEAP internal control, driven by a CMV promoter using a commercially available plasmid (GeneCopoeia, Rockville, MD; Fig. 1A). Luciferase-expressing cells were incubated with 2007 FDA-approved drugs from NIH Clinical Drug Collections NCC-202 and NCC-105 (727 compounds) and the MicroSource Spectrum Collection (1280 compounds) libraries for 24 hours at 3 μM concentration, and media collected for luciferase and SEAP analysis (Fig. 1B).

To identify hits from the screen, the luciferase/SEAP signal was calculated for each drug, and normalized to DMSO-treated control in the absence of any drug, as described in the Methods. The mean DMSO-treated control luciferase/SEAP level in the absence of any drugs was set as 1. The mean + SD for luciferase/SEAP values across all drugs in the screen normalized to DMSO control was 1.0231 + 0.1232, or 2.31 + 12.32% increase from DMSO control. From this, we calculated the mean + 3SD of the screen, a commonly used cut-off level in drug screen evaluations to identify hits [47–49], to be 1.3926, or 39.26% increase from the mean DMSO luciferase/SEAP value for all drugs in the screen, which resulted in 18 unique hits (Fig. 1B, Fig. S1A).

Secondary assay carried out by RT-qPCR in human Eahy926 endothelial cell line revealed that most of the hits from initial screening in HEK293t cells did not significantly upregulate *PICALM* mRNA relative to vehicle 24 hours after drug application (Fig. 1C). Some compounds were not tested because of reported neuronal toxicity in the literature (see Fig. S1B). The hits that showed significant *PICALM* mRNA upregulation in Eahy926 cells were the anti-malaria drug artesunate and antimetabolite anti-psoriatic drug 6-azauridine (Fig. 1C; Fig. S1B). Of these two drugs, 6-azauridine had been removed from clinical use due to potential thrombotic complications [67], and possible carcinogenicity and neurotoxicity (Fig. S1B). This yielded artesunate as the lead drug candidate.

Further testing showed that incubation of Eahy926 cells with artesunate (3 μM for 24 h) upregulated both *PICALM* mRNA and PICALM protein levels by approximately 2-fold (Fig. 1D, E), and that artesunate dose-dependently upregulated *PICALM* mRNA with EC50 of 2.1 μM (Fig. 1F). Since it had been reported that EGR1 transcription factor controls PICALM expression [68], we next studied whether artesunate requires EGR1 to increase PICALM. Our data show that artesunate upregulated *EGR1* mRNA in Eahy926 cells by approximately 2-fold (Fig. 1G), and that silencing *EGR1* abolished artesunate-mediated PICALM upregulation (Fig. 1H), indicating that EGR1 is required for artesunate-mediated PICALM upregulation.

To see whether artesunate upregulates PICALM in vivo, we treated *Picalm*-deficient (*Picalm*^+/−^) mice [28, 53] with a low artesunate dose (32 mg/kg i.p.) for 7 days as previously reported [58]. Compared to vehicle, artesunate increased PICALM protein levels in brain capillaries of *Picalm*^+/−^ mice by 2-fold (Fig. 1I), but did not significantly alter PICALM levels in capillary-depleted brains (Fig. 1J). These data suggest that artesunate upregulates PICALM in the mouse brain capillaries in vivo confirming our in vitro observations in the human endothelial cell line.

### Artesunate slows development of Aβ pathology and functional decline in a *Picalm*-deficient mouse model of AD

To create a mouse model that recapitulates features of PICALM reduction in AD [28, 31, 32], we crossed *Picalm*^+/−^ mice [28, 53] to the *5XFAD* mouse line [54] and generated *Picalm*-deficient *Picalm*^+/−^*; 5XFAD* model with elevated Aβ pathology compared to age-matched *5XFAD* littermates as shown by increased Aβ42 and Aβ40 levels in the hippocampus and cortex by 78 and 64% (Aβ42), and 160 and 130% (Aβ40), respectively, and increased Aβ load and thioflavin S-positive plaque load in the hippocampus and cortex by 78 and 67% (Aβ load), and 101 and 108% (thioflavin S load), respectively (Fig. S2). However, we did not find differences in amyloid precursor protein (APP) processing, as shown by comparable brain levels of APP, APP C-terminal fragment (APP-CTF), β-secretase (BACE1) and Notch intracellular domain (NICD) fragment from Notch protein reflecting its production by γ–secretase, or differences in Aβ degrading enzymes, as shown by comparable brain levels of neprilysin and insulin-degrading enzyme (IDE), similar to that reported previously in *Picalm*^+/−^ crosses with *APP*^sw/0^ mice [28] (Fig. S2).

Starting at 3 months of age, we treated *Picalm*^+/−^; *5XFAD* mice with i.p. artesunate (32 mg/kg/day) or vehicle for 2 months (Fig. 2A). Analysis of brain capillaries isolated from these mice revealed a 2-fold increase in PICALM levels after artesunate treatment (Fig. 2B). Consistent with previously shown role of PICALM in enhancing Aβ clearance across the BBB in vivo [28], artesunate treatment compared to vehicle reduced Aβ42 and Aβ40 levels in the cortex and hippocampus of *Picalm*^+/−^; *5XFAD* mice by 42 and 44%, and 29 and 33%, respectively (Fig. 2C, D). Similarly, artesunate reduced Aβ load and thioflavin S-positive amyloid in the cortex and hippocampus by 34–51% (Fig. 2E-H) and amyloid accumulation in in small pial arteries and penetrating cortical arteriole blood vessels by 44% (Fig. 2I, J). Importantly, artesunate treatment increased blood serum Aβ42 and Aβ40 levels by approximately 2-fold suggesting accelerated clearance of Aβ from brain-to-blood (Fig. 2K, L).

Consistent with the reduction in Aβ pathology, artesunate treatment of *Picalm*^+/−^; *5XFAD* mice improved cerebral blood flow (CBF) response to whisker stimulation by 53% (Fig. 2M), reduced leakage of blood-derived fibrinogen across the BBB by 27% (Fig. 2N, O), and improved behavioral performance on novel object location, novel object recognition, burrowing and nesting tests (Fig. 2P-S).

Artesunate also suppressed the neuroinflammatory response, as indicated by significantly (*p* < 0.05) lower numbers of ionized calcium-binding adapter molecule 1 (Iba1)-positive microglia by 28–25% and glial fibrillar acidic protein (GFAP)-positive astrocytes by 23–36% in the hippocampus and cortex, respectively, of *Picalm*^+/−^; *5XFAD* mice (Fig. S3A-D).

Evaluation of biochemical parameters in blood indicated no differences between artesunate- and vehicle-treated *Picalm*^+/−^; *5XFAD* mice confirming normal levels of liver enzymes, normal kidney analytes, no increase in enzymes reflecting heart and skeletal muscle damage and normal glucose levels, and no differences in bodyweight over the course of treatment (Fig. S4). These data suggest that artesunate does not exert systemic effects that could potentially influence its central action.

Importantly, artesunate treatment did not alter amyloid precursor protein (APP) processing or Aβ degrading enzymes. This has been shown by comparable brain levels of APP and BACE1 and CSF levels of soluble APP-β (sAPPβ), as well as by comparable brain levels of NICD fragment from Notch protein reflecting its production by γ–secretase, as well as by comparable levels of neprilysin and IDE in both artesunate- and vehicle-treated mice (Fig. 3).

### Deletion of endothelial *Picalm* abolishes the beneficial effects of artesunate

To confirm more directly the role of endothelial PICALM in the observed beneficial effects of artesunate, we next generated endothelial-specific *Picalm* knockout mice by crossing cadherin 5 (Cdh5)-Cre mice with mice carrying floxed exon 2 of the *Picalm* gene [53]. As shown by immunostaining for PICALM and endothelial-specific lectin in brain capillary cytospins prepared from *Picalm*^lox/lox^; *Cdh5*-Cre mice and *Picalm*^lox/lox^ control littermates, PICALM was nearly undetectable by immunostaining in capillary endothelium from *Picalm*^lox/lox^; *Cdh5*-Cre mice compared to its robust expression in endothelium in control *Picalm*^lox/lox^ littermates (Fig. 4A). Deletion of PICALM from endothelium in *Picalm*^lox/lox^; *Cdh5*-Cre was also confirmed by immunoblotting of brain endothelial cells cultured from brain capillaries of these mice showing barely detectable PICALM band compared to endothelial cells from *Picalm*^lox/lox^ littermates (Fig. 4B). Additionally, we also show that brain capillaries from *Picalm*^lox/lox^; *Cdh5*-Cre mice compared to *Picalm*^lox/lox^ controls had barely detectable PICALM levels (Fig. 4C), while PICALM levels were unchanged in capillary depleted brain of these mice (Fig. 4D).

We then crossed *Picalm*^lox/lox^; *Cdh5*-Cre mice to *5XFAD* mice to generate *Picalm*^lox/lox^; *Cdh5*-Cre; *5XFAD* mice with a complete endothelial-specific *Picalm* deletion. Using the artesunate treatment protocol as for the *Picalm*^+/−^; *5XFAD* animals above, at 3 months of age, we treated *Picalm*^lox/lox^; *Cdh5*-Cre; *5XFAD* mice with i.p. artesunate (32 mg/kg/day) or vehicle for 2 months (Fig. 4E). We found that artesunate failed to reduce Aβ pathology in hippocampus or cortex of *Picalm*^lox/lox^; *Cdh5*-Cre; *5XFAD* mice as indicated by comparable Aβ42 or Aβ40 levels (Fig. 4F, G), Aβ load and thioflavin S-positive amyloid load (Fig. 4H-K) in artesunate-treated compared to vehicle-treated 5XFAD mice lacking endothelial PICALM. Moreover, artesunate compared to vehicle did not reduce amyloid load in blood vessels (Fig. 4L, M), nor improved CBF responses to whisker stimulus (Fig. 4N) or performance on four behavior tests in *Picalm*^lox/lox^; *Cdh5*-Cre; *5XFAD* (Fig. 4O-R). Altogether these data indicate that endothelial PICALM is critical for the observed beneficial effects of artesunate on Aβ clearance and pathology, and the associated functional outcome measures.

## Discussion

Our screening of 2007 FDA-approved drugs in HEK293t cells expressing luciferase followed by a secondary mRNA screen in the human Eahy296 endothelial cell line identified anti-malaria drug artesunate as the lead hit. We showed that artesunate elevated by 2–3-fold PICALM mRNA and protein levels in the Eahy296 cell line, and in vivo in brain capillaries isolated from artesunate-treated *Picalm*^*+/−*^ mice. We next showed that artesunate treatment of *Picalm*^*+/−*^; *5XFAD* mice increased brain capillary PICALM levels by 2-fold, and reduced by 33–51% Aβ42 and Aβ40 levels, Aβ load, and thioflavin S-positive amyloid in brain, as well as amyloid accumulation in small pial arteries and penetrating cortical arterioles representing CAA. The effects of artesunate on Aβ pathology were associated with improved functional outcome including improved CBF responses, BBB integrity and behavior on four different behavioral tests.

The observed beneficial effects of artesunate involved upregulation of PICALM in brain capillaries, a site of the BBB in vivo [69, 70], consistent with previous findings showing that PICALM has a central role in controlling Aβ transcytosis and clearance across the BBB [28]. This role of PICALM includes regulation of key molecular steps controlling Aβ trans-endothelial transport including clathrin-dependent endocytosis of Aβ-LRP1 complexes at the abluminal side of the BBB endothelium followed by guidance of internalized Aβ-LRP1 vesicles to Rab5 early endosomes [26] and then to Rab11, a GTPase which regulates recycling of vesicles controlling transcytosis [71–73] and exocytosis [74] of ligands, ultimately resulting in Aβ transcytosis and clearance across the BBB.

Although artesunate can reportedly cross the BBB [75] which potentially may affect cells in the brain parenchyma directly, in our hands artesunate treatment did not influence processing of Aβ protein as shown by normal levels of APP, BACE1 and γ-secretase activity in brain tissue, and normal production of sAPPβ in the CSF [76]. Artesunate also did not affect the levels of Aβ degrading enzymes neprilysin and IDE [76] in brain tissue. Additionally, we did not find an increase in PICALM levels in capillary-depleted brains from *Picalm*^*+/−*^ mice which altogether suggests that the primary mechanism of artesunate action in *Picalm*^*+/−*^; *5XFAD* mice is upregulation of PICALM in endothelium that in turn leads to enhanced Aβ clearance across the BBB. This is supported by our finding of substantially higher levels of Aβ42 and Aβ40 in serum of artesunate-treated animals compared to vehicle reflecting accelerated brain-to-blood clearance of Aβ.

Several previous studies [28, 36, 37] and the present study suggest that loss of PICALM increases Aβ pathology [28, 36] and/or tau pathology [37], and leads to loss of neuronal protection against Aβ toxicity [27, 77]. However, it has also been reported that reduction in PICALM may result in reduced internalization of γ-secretase which in turn may reduce Aβ pathology as shown in older *Picalm*-deficient *Picalm*^*+/−*^; *A7* mice expressing human APP with the Swedish and Austrian familial AD mutations [78].

Although we do not have an explanation for the discrepancy between the present and previous studies [27, 28, 36, 77] compared to a study showing reduction of Aβ pathology in the presence of PICALM deficiency [78], it is possible that choice of model, experimental parameters, or other factors may contribute to the differences observed. For example, notable differences between *Picalm*-deficient *A7* mice compared to *5XFAD* model of *Picalm* deficiency are that *A7* mice develop pathology at a much slower rate than *5XFAD* mice with Aβ42/Aβ40 ratio of over 100:1, in contrast to *5XFAD* mice with five familial AD mutations (3 in APP: Swedish, Florida, London; and 2 in presenilin 1 (PS1): M146L+ L286V), that develop Aβ pathology much earlier with Aβ42/Aβ40 ratio typically on the order of 10–20:1 [50, 54, 79]. It is possible that the relative contributions of PICALM to different processes involved in Aβ homeostasis in different models may lead to predominant activation of the clearance mechanism across the BBB as we and others see in *Picalm*-deficient *5XFAD* model as opposed to effects on APP processing in neurons as seen in *Picalm*-deficient A7 model.

To further confirm the role of endothelial PICALM in mediating beneficial effects of artesunate, we next studied the effects of artesunate treatment on Aβ pathology and functional outcome in *Picalm*^lox/lox^; *Cdh5*-Cre; *5XFAD* mice with a complete endothelial-specific *Picalm* deletion. Our data showed that PICALM is almost completely deleted from brain capillary endothelium in *Picalm*^lox/lox^; *Cdh5*-Cre mice compared to its robust expression in control *Picalm*^lox/lox^ mice. We also showed that brain capillaries isolated from *Picalm*^lox/lox^; *Cdh5*-Cre mice have barely detectable PICALM levels compared to their *Picalm*^lox/lox^ littermate controls. This not only suggests that PICALM was efficiently deleted from the BBB of *Picalm*^lox/lox^; *Cdh5*-Cre mice but also indicates that endothelial PICALM is a major source of brain capillary PICALM in mice. Next, we showed that lack of endothelial PICALM abolished all beneficial effects of artesunate in *5XFAD* mice including those lowering Aβ pathology in brain as well as CAA. Consistent with these findings, artesunate failed to improve CBF responses and behavioral deficits in *Picalm*^lox/lox^; *Cdh5*-Cre; *5XFAD* mice, overall confirming that endothelial PICALM is critical for the observed therapeutic effects of artesunate on Aβ pathology and functional outcome.

Artesunate, a soluble derivative of artemisinin, is currently in widespread use around the world to treat the parasitic infection malaria [80], and is thought to function in this capacity by blocking cytochrome oxidase in the parasites and producing reactive oxides in erythrocytes, causing parasitic death [81]. Recent interest in artesunate because of its potential anti-cancer, anti-inflammation, and vascular injury applications [80–83] also revealed that artesunate may act via alternate pathways in non-parasitic conditions. However, little is known regarding mechanisms of PICALM regulation. Consistent with a previous report suggesting that the transcription factor EGR1 regulates *PICALM* expression, and that increase in EGR1 expression led to increased luciferase reporter expression driven by a proximal *PICALM* promotor [68], we also found that artesunate upregulated *EGR1* mRNA in the endothelial Eahy926 cell line, and that silencing *EGR1* abolished artesunate-mediated upregulation of PICALM. These data suggest that EGR1 is required for the observed artesunate-mediated upregulation of PICALM. Artesunate was also reported to activate the PI3K/Akt pathway in rodent models of subarachnoid hemorrhage and myocardial infarction [82, 83], and the PI3K/Akt pathway has also been shown to control EGR1 expression [84]. Whether these pathways reflect an upstream mechanism of PICALM upregulation by artesunate, and whether the same mechanisms can operate in brain endothelium in vivo remain to be determined by future studies.

Few clinical trials have investigated the potential side effects of long-term treatment with artesunate or artesunate-like compounds. A clinical study to evaluate the efficacy of artemisinin, a variant of artesunate, for treating schizophrenia reported no adverse effects over placebo after 8 weeks of treatment [85]. This suggests that artesunate is likely well tolerated over longer periods in humans, but further study is required to develop and optimize its regimen in patients with cognitive impairment diagnosed within the AD spectrum.

## Conclusions

The present results further underscore the importance of vascular function and Aβ clearance across the BBB in preventing the progression of AD-like pathology and slowing functional decline. Our treatment was successful in animals with relatively early stage Aβ pathology, but remains to be seen whether artesunate can also be beneficial in the *5XFAD* model at a later stage with full-blown pathology and cognitive impairment [54], and by extension in individuals with mid- to late-stage AD when amyloid accumulation is higher. Nevertheless, the present data supports the potential of artesunate for early stage disease in AD patients with positive Aβ biomarkers including those with pre-clinical cognitive impairment and early-stages of clinical progression to AD.

## Supplementary Information

**Additional file 1: Fig. S1.** Drug hits identified with the HEK293t luciferase reporter assay and a secondary RT-qPCR *PICALM* mRNA screen in Eahy926 endothelial cells. (**A**) Unique hits identified by the PICALM; Luciferase screen in HEK293t cells shown in Fig. 1B. Percent increase of luciferase luminescence from screen, normalized to SEAP and DMSO control (See Fig. 1A, B and Methods) are indicated. (**B**) Table summarizing results of RT-qPCR *PICALM* mRNA screen in Eahy926 endothelial cells shown in Fig. 1C, and description of toxic or adverse indications reported in literature. Picrotoneoamine and corticosterone were not evaluated further because of their close similarity to other drug hits that did not yield significant *PICALM* mRNA increases and their reported toxicity.

**Additional file 2: Fig. S2.** Characterization of PICALM loss and amyloid pathology in *Picalm*^+/−^; *5XFAD* mice compared to *5XFAD* littermates. (**A**) PICALM protein relative expression levels in cortex isolated from *5XFAD* mice or littermate *Picalm*^+/−^; *5XFAD* mice treated with vehicle as in Fig. 2A. (**B, C**) Amyloid-β 42 (Aβ42) (**B**) and Aβ40 (**C**) levels in the hippocampus (Hpp) and cortex (Ctx) in *5XFAD* mice or littermate *Picalm*^+/−^; *5XFAD* mice treated with vehicle. *Picalm*^+/−^; *5XFAD* data is replotted from Fig. 2C, D. (**D**) Quantification of Aβ load from pan-Aβ immunostaining in the Hpp and Ctx in *5XFAD* mice or littermate *Picalm*^+/−^; *5XFAD* mice treated with vehicle. *Picalm*^+/−^; *5XFAD* data is replotted from Fig. 2F. (**E**) Quantification of Thioflavin S amyloid plaque load in the Hpp and Ctx in *5XFAD* mice or *Picalm*^+/−^; *5XFAD* mice treated with vehicle. *Picalm*^+/−^; *5XFAD* data is replotted from Fig. 2H. (**F-K**) Western immunoblotting of Aβ processing proteins and Aβ clearance enzymes. (**F**) amyloid precursor protein (APP) abundance in cortex, (**G**) APP C-terminal fragment (APP-CTF) abundance in cortex. (**H**) β-secretase (BACE1) abundance in cortex. (**I**) γ–secretase activity as determined by the production of Notch intracellular domain (NICD) fragment from Notch protein, indicated by NICD abundance in cortex, (**J**) neprilysin abundance in cortex, and (**K**) insulin degrading enzyme (IDE) abundance in cortex of *5XFAD* mice or littermate *Picalm*^+/−^; *5XFAD* mice treated with vehicle. The relative abundance of proteins was normalized by the house-keeping gene glyceraldehyde 3-phosphate dehydrogenase (GAPDH) protein. Data is single points per mouse indicated by circles**,** with mean ± SD; *n* = 7 mice per condition. Significance determined by Student’s two-tail t-test. ns = non-significant by two-tailed t-test. Full blots for A, F-K shown in supp. Fig. 5.

**Additional file 3: Fig. S3.** Artesunate alleviates microglia and astrocyte responses *in Picalm*^+/−^; *5XFAD* mice. (**A,B**) Representative images of Iba1-positive microglia (red) and Thioflavin S-positive amyloid deposits (ThS, green) in cortex (**A**), and quantification of Iba1-positive microglia in the hippocampus (Hpp) and cortex (Ctx) (**B**) in *Picalm*^+/−^; *5XFAD* mice treated with vehicle or artesunate (Art) as shown in Fig. 2A. (**C,D**) Representative images of GFAP-positive astrocytes (red) and Thioflavin S-positive amyloid deposits (green) in cortex (**C**), and quantification of GFAP-positive astrocytes in the Hpp and Ctx (**D**) in *Picalm*^+/−^; *5XFAD* mice treated with vehicle or Art as shown in Fig. 2A. Scale bars in **A, C** are 10 μm. *n* = 8 mice per condition. Single points per mouse indicated by circles in **B, D,** with mean ± SD. Significance determined by Student’s two-tail t-test in panels **B**, **D**.

**Additional file 4: Fig. S4.** Biochemical parameters in blood show no differences between artesunate- and vehicle-treated *Picalm*^+/−^; *5XFAD* mice. *Picalm*^+/−^; *5XFAD* mice were treated with vehicle or artesunate (Art) as in Fig. 2A. (**A**) Alkaline phosphatase (ALP), (**B**) aspartate aminotransferase (AST), (**C**) alanine aminotransferase (ALT), (**D**) albumin, (**E**) total protein, (**F**) total bilirubin, (**G**) blood urea nitrogen, (**H**) calcium, (**I**) creatine kinase, and (**J**) glucose. (**K**) Mouse weights normalized to treatment starting weight for *Picalm*^+/−^; *5XFAD* mice treated for two months with vehicle or Art starting at 3 mo of age as in Fig. 2A. **A**,**B**, **D-J**, *n* = 11 mice per condition; **C**, *n* = 9 vehicle and 11 Art treated mice; **K**, *n* = 14 vehicle and 15 Art treated mice. **A-J**, data is single points per mouse indicated by circles in with mean ± SD. In **K**, data is mean ± SEM. **A-E**, **G-J**, statistical significance was determined by Student’s t-test, **F** by Mann-Whitney U test, and **K** by two-way ANOVA followed by Bonferroni post-test. ns = non-significant.

**Additional file 5: Fig. S5.** Full Western immunoblots for data shown in Figs. 1, 2, 3 and 4 and Supp. Figure 2. Blots for each figure are indicated. Dashed lines indicate bands used as representative images. Blots for Figs. 1, 2, and 4 were imaged with CL-XPosure film, using loading amounts and exposures within the linear dynamic range of the film. Blots for Fig. 3 and S2 were imaged with a Carestream IS4000MM Pro Image Station digital chemiluminescence gel detection instrument within the linear dynamic range of the instrument (see Methods for blotting and detection details).

## Data Availability

The datasets used and/or analyzed during the current study are available from the corresponding author on reasonable request.

## Abbreviations

AD: Alzheimer’s disease
Art: Artesunate
APP: Amyloid precursor protein
Aβ: Amyloid-β
αSMA: α-smooth muscle actin
BACE1: β-secretase
BBB: Blood-brain barrier
CAA: Cerebral amyloid angiopathy
CBF: Cerebral blood flow
Cdh5: Cadherin 5
Ctx: Cortex
DMSO: Dimethyl sulfoxide
EGR1: Early growth response 1
GAPDH: Gene glyceraldehyde 3-phosphate dehydrogenase
GFAP: Glial fibrillar acidic protein
Hpp: Hippocampus
Iba1: Ionized calcium-binding adapter molecule 1
IDE: Insulin-degrading enzyme
iPSC: Induced pluripotent stem cells
LOAD: Late onset Alzheimer’s disease
LRP1: Lipoprotein receptor related protein 1
NICD: Notch intracellular domain
NOL: Novel object location
NOR: Novel object recognition
PICALM: Phosphatidylinositol binding clathrin assembly protein
RT-qPCR: Real-time quantitative polymerase chain reaction
sAPPβ: Soluble APPβ
SEAP: Secreted alkaline phosphatase

## References

[1] Dreyling MH, Martinez-Climent JA, Zheng M, Mao J, Rowley JD, Bohlander SK (1996). The t(10;11)(p13;q14) in the U937 cell line results in the fusion of the AF10 gene and CALM, encoding a new member of the AP-3 clathrin assembly protein family. Proc Natl Acad Sci U S A.

[2] Tebar F, Bohlander SK, Sorkin A (1999). Clathrin assembly lymphoid myeloid leukemia (CALM) protein: localization in endocytic-coated pits, interactions with clathrin, and the impact of overexpression on clathrin-mediated traffic. Mol Biol Cell.

[3] Harold D, Abraham R, Hollingworth P, Sims R, Gerrish A, Hamshere ML (2009). Genome-wide association study identifies variants at CLU and PICALM associated with Alzheimer’s disease. Nat Genet.

[4] Lambert J-C, Heath S, Even G, Campion D, Sleegers K, Hiltunen M (2009). Genome-wide association study identifies variants at CLU and CR1 associated with Alzheimer’s disease. Nat Genet.

[5] Carrasquillo MM, Belbin O, Hunter TA, Ma L, Bisceglio GD, Zou F, et al. Replication of CLU, CR1, and PICALM Associations With Alzheimer Disease. Arch Neurol. 2010;67 [cited 2021 Apr 24]. Available from: http://archneur.jamanetwork.com/article.aspx?doi=10.1001/archneurol.2010.147. 10.1001/archneurol.2010.147 PMC2919638 20554627

[6] Lambert J-C, Zelenika D, Hiltunen M, Chouraki V, Combarros O, Bullido MJ (2011). Evidence of the association of BIN1 and PICALM with the AD risk in contrasting European populations. Neurobiol Aging.

[7] Schjeide B-MM, Schnack C, Lambert J-C, Lill CM, Kirchheiner J, Tumani H (2011). The role of clusterin, complement receptor 1, and phosphatidylinositol binding clathrin assembly protein in Alzheimer disease risk and cerebrospinal fluid biomarker levels. Arch Gen Psychiatry.

[8] Naj AC, Jun G, Beecham GW, Wang L-S, Vardarajan BN, Buros J (2011). Common variants at MS4A4/MS4A6E, CD2AP, CD33 and EPHA1 are associated with late-onset Alzheimer’s disease. Nat Genet.

[9] Chen LH, Kao PYP, Fan YH, Ho DTY, Chan CSY, Yik PY (2012). Polymorphisms of CR1, CLU and PICALM confer susceptibility of Alzheimer’s disease in a southern Chinese population. Neurobiol Aging.

[10] Liu G, Zhang S, Cai Z, Ma G, Zhang L, Jiang Y (2013). PICALM gene rs3851179 polymorphism contributes to Alzheimer’s disease in an Asian population. NeuroMolecular Med.

[11] Lambert JC, Ibrahim-Verbaas CA, Harold D, Naj AC, Sims R, Bellenguez C (2013). Meta-analysis of 74,046 individuals identifies 11 new susceptibility loci for Alzheimer’s disease. Nat Genet.

[12] Morgen K, Ramirez A, Frölich L, Tost H, Plichta MM, Kölsch H (2014). Genetic interaction of *PICALM* and *APOE* is associated with brain atrophy and cognitive impairment in Alzheimer’s disease. Alzheimers Dement.

[13] Gharesouran J, Rezazadeh M, Khorrami A, Ghojazadeh M, Talebi M (2014). Genetic evidence for the involvement of variants at APOE, BIN1, CR1, and PICALM loci in risk of late-onset Alzheimer’s disease and evaluation for interactions with APOE genotypes. J Mol Neurosci.

[14] Belcavello L, Camporez D, Almeida LD, Morelato RL, Batitucci MCP, de Paula F (2015). Association of MTHFR and PICALM polymorphisms with Alzheimer’s disease. Mol Biol Rep.

[15] Huang K-L, Marcora E, Pimenova AA, Di Narzo AF, Kapoor M, Jin SC (2017). A common haplotype lowers PU.1 expression in myeloid cells and delays onset of Alzheimer’s disease. Nat Neurosci.

[16] Santos-Rebouças CB, Gonçalves AP, Dos Santos JM, Abdala BB, Motta LB, Laks J (2017). rs3851179 polymorphism at 5′ to the PICALM gene is associated with Alzheimer and Parkinson diseases in Brazilian population. NeuroMolecular Med.

[17] Sun D-M, Chen H-F, Zuo Q-L, Su F, Bai F, Liu C-F (2017). Effect of PICALM rs3851179 polymorphism on the default mode network function in mild cognitive impairment. Behav Brain Res.

[18] Raj T, Li YI, Wong G, Humphrey J, Wang M, Ramdhani S (2018). Integrative transcriptome analyses of the aging brain implicate altered splicing in Alzheimer’s disease susceptibility. Nat Genet.

[19] Kunkle BW, Grenier-Boley B, Sims R, Bis JC, Damotte V, Naj AC (2019). Genetic meta-analysis of diagnosed Alzheimer’s disease identifies new risk loci and implicates Aβ, tau, immunity and lipid processing. Nat Genet.

[20] Jansen IE, Savage JE, Watanabe K, Bryois J, Williams DM, Steinberg S (2019). Genome-wide meta-analysis identifies new loci and functional pathways influencing Alzheimer’s disease risk. Nat Genet.

[21] Zeng F-F, Liu J, He H, Gao X-P, Liao M-Q, Yu X-X (2019). Association of PICALM gene polymorphisms with Alzheimer’s disease: evidence from an updated Meta-analysis. Curr Alzheimer Res.

[22] Masri I, Salami A, El Shamieh S, Bissar-Tadmouri N (2020). rs3851179G>a in PICALM is protective against Alzheimer’s disease in five different countries surrounding the Mediterranean. Curr Aging Sci..

[23] Juul Rasmussen I, Tybjærg-Hansen A, Rasmussen KL, Nordestgaard BG, Frikke-Schmidt R (2019). Blood–brain barrier transcytosis genes, risk of dementia and stroke: a prospective cohort study of 74,754 individuals. Eur J Epidemiol.

[24] Marsh M, McMahon HT (1999). The structural era of endocytosis. Science..

[25] Ford MG, Pearse BM, Higgins MK, Vallis Y, Owen DJ, Gibson A (2001). Simultaneous binding of PtdIns (4,5) P2 and clathrin by AP180 in the nucleation of clathrin lattices on membranes. Science..

[26] Sorkin A, von Zastrow M (2009). Endocytosis and signalling: intertwining molecular networks. Nat Rev Mol Cell Biol.

[27] Treusch S, Hamamichi S, Goodman JL, Matlack KES, Chung CY, Baru V (2011). Functional links between Aβ toxicity, endocytic trafficking, and Alzheimer’s disease risk factors in yeast. Science..

[28] Zhao Z, Sagare AP, Ma Q, Halliday MR, Kong P, Kisler K (2015). Central role for PICALM in amyloid-β blood-brain barrier transcytosis and clearance. Nat Neurosci.

[29] Vecchi M, Polo S, Poupon V, van de Loo JW, Benmerah A, Fiore D (2001). Nucleocytoplasmic shuttling of endocytic proteins. J Cell Biol.

[30] Miller SE, Sahlender DA, Graham SC, Höning S, Robinson MS, Peden AA (2011). The molecular basis for the endocytosis of small R-SNAREs by the clathrin adaptor CALM. Cell..

[31] Baig S, Joseph SA, Tayler H, Abraham R, Owen MJ, Williams J (2010). Distribution and expression of picalm in Alzheimer disease. J Neuropathol Exp Neurol.

[32] Parikh I, Fardo DW, Estus S (2014). Genetics of PICALM expression and Alzheimer’s disease. PLoS One.

[33] Ando K, Brion J-P, Stygelbout V, Suain V, Authelet M, Dedecker R (2013). Clathrin adaptor CALM/PICALM is associated with neurofibrillary tangles and is cleaved in Alzheimer’s brains. Acta Neuropathol.

[34] Kanatsu K, Morohashi Y, Suzuki M, Kuroda H, Watanabe T, Tomita T (2014). Decreased CALM expression reduces Aβ42 to total Aβ ratio through clathrin-mediated endocytosis of γ-secretase. Nat Commun.

[35] Zhang Y, Sloan SA, Clarke LE, Caneda C, Plaza CA, Blumenthal PD (2016). Purification and characterization of progenitor and mature human astrocytes reveals transcriptional and functional differences with Mouse. Neuron..

[36] Tian Y, Chang JC, Fan EY, Flajolet M, Greengard P (2013). Adaptor complex AP2/PICALM, through interaction with LC3, targets Alzheimer’s APP-CTF for terminal degradation via autophagy. Proc Natl Acad Sci U S A.

[37] Ando K, De Decker R, Vergara C, Yilmaz Z, Mansour S, Suain V (2020). Picalm reduction exacerbates tau pathology in a murine tauopathy model. Acta Neuropathol.

[38] Rauch JN, Luna G, Guzman E, Audouard M, Challis C, Sibih YE (2020). LRP1 is a master regulator of tau uptake and spread. Nature..

[39] Shibata M, Yamada S, Kumar SR, Calero M, Bading J, Frangione B (2000). Clearance of Alzheimer’s amyloid-ss (1-40) peptide from brain by LDL receptor-related protein-1 at the blood-brain barrier. J Clin Invest.

[40] Deane R, Wu Z, Sagare A, Davis J, Du Yan S, Hamm K (2004). LRP/amyloid beta-peptide interaction mediates differential brain efflux of Abeta isoforms. Neuron..

[41] Bell RD, Sagare AP, Friedman AE, Bedi GS, Holtzman DM, Deane R (2007). Transport pathways for clearance of human Alzheimer’s amyloid beta-peptide and apolipoproteins E and J in the mouse central nervous system. J Cereb Blood Flow Metab.

[42] Deane R, Sagare A, Hamm K, Parisi M, Lane S, Finn MB (2008). apoE isoform-specific disruption of amyloid beta peptide clearance from mouse brain. J Clin Invest.

[43] Jaeger LB, Dohgu S, Sultana R, Lynch JL, Owen JB, Erickson MA (2009). Lipopolysaccharide alters the blood-brain barrier transport of amyloid beta protein: a mechanism for inflammation in the progression of Alzheimer’s disease. Brain Behav Immun.

[44] Hong H, Liu LP, Liao JM, Wang TS, Ye FY, Wu J (2009). Downregulation of LRP1 [correction of LPR1] at the blood-brain barrier in streptozotocin-induced diabetic mice. Neuropharmacology..

[45] Sagare AP, Bell RD, Zhao Z, Ma Q, Winkler EA, Ramanathan A (2013). Pericyte loss influences Alzheimer-like neurodegeneration in mice. Nat Commun.

[46] Winkler EA, Nishida Y, Sagare AP, Rege SV, Bell RD, Perlmutter D (2015). GLUT1 reductions exacerbate Alzheimer’s disease vasculo-neuronal dysfunction and degeneration. Nat Neurosci.

[47] Malo N, Hanley JA, Cerquozzi S, Pelletier J, Nadon R (2006). Statistical practice in high-throughput screening data analysis. Nat Biotechnol.

[48] Shun TY, Lazo JS, Sharlow ER, Johnston PA (2011). Identifying actives from HTS data sets: practical approaches for the selection of an appropriate HTS data-processing method and quality control review. J Biomol Screen.

[49] Aldewachi H, Al-Zidan RN, Conner MT, Salman MM (2021). High-throughput screening platforms in the discovery of novel drugs for neurodegenerative diseases. Bioengineering (Basel).

[50] Lazic D, Sagare AP, Nikolakopoulou AM, Griffin JH, Vassar R, Zlokovic BV (2019). 3K3A-activated protein C blocks amyloidogenic BACE1 pathway and improves functional outcome in mice. J Exp Med.

[51] Montagne A, Nikolakopoulou AM, Zhao Z, Sagare AP, Si G, Lazic D (2018). Pericyte degeneration causes white matter dysfunction in the mouse central nervous system. Nat Med.

[52] Guo H, Zhao Z, Yang Q, Wang M, Bell RD, Wang S (2013). An activated protein C analog stimulates neuronal production by human neural progenitor cells via a PAR1-PAR3-S1PR1-Akt pathway. J Neurosci.

[53] Ishikawa Y, Maeda M, Pasham M, Aguet F, Tacheva-Grigorova SK, Masuda T (2015). Role of the clathrin adaptor PICALM in normal hematopoiesis and polycythemia vera pathophysiology. Haematologica..

[54] Oakley H, Cole SL, Logan S, Maus E, Shao P, Craft J (2006). Intraneuronal beta-amyloid aggregates, neurodegeneration, and neuron loss in transgenic mice with five familial Alzheimer’s disease mutations: potential factors in amyloid plaque formation. J Neurosci.

[55] Devi L, Tang J, Ohno M (2015). Beneficial effects of the β-secretase inhibitor GRL-8234 in 5XFAD Alzheimer’s transgenic mice lessen during disease progression. Curr Alzheimer Res.

[56] Devi L, Ohno M (2015). Effects of BACE1 haploinsufficiency on APP processing and Aβ concentrations in male and female 5XFAD Alzheimer mice at different disease stages. Neuroscience..

[57] Mariani MM, Malm T, Lamb R, Jay TR, Neilson L, Casali B (2017). Neuronally-directed effects of RXR activation in a mouse model of Alzheimer’s disease. Sci Rep.

[58] Clemmer L, Martins YC, Zanini GM, Frangos JA, Carvalho LJM (2011). Artemether and artesunate show the highest efficacies in rescuing mice with late-stage cerebral malaria and rapidly decrease leukocyte accumulation in the brain. Antimicrob Agents Chemother.

[59] Bell RD, Winkler EA, Sagare AP, Singh I, LaRue B, Deane R (2010). Pericytes control key neurovascular functions and neuronal phenotype in the adult brain and during brain aging. Neuron..

[60] Sagare AP, Bell RD, Srivastava A, Sengillo JD, Singh I, Nishida Y (2013). A lipoprotein receptor cluster IV mutant preferentially binds amyloid-β and regulates its clearance from the mouse brain. J Biol Chem.

[61] Deane R, Singh I, Sagare AP, Bell RD, Ross NT, LaRue B (2012). A multimodal RAGE-specific inhibitor reduces amyloid β-mediated brain disorder in a mouse model of Alzheimer disease. J Clin Invest.

[62] Wu Z, Hofman FM, Zlokovic BV (2003). A simple method for isolation and characterization of mouse brain microvascular endothelial cells. J Neurosci Methods.

[63] Mackic  JB, Stins  M, McComb JG, Calero  M, Ghiso  J, Kim  KS (1998). Human blood-brain barrier receptors for Alzheimer’s amyloid-beta 1–40. Asymmetrical binding, endocytosis, and transcytosis at the apical side of brain microvascular endothelial cell monolayer. J Clin Invest.

[64] Cortes-Canteli M, Paul J, Norris EH, Bronstein R, Ahn HJ, Zamolodchikov D (2010). Fibrinogen and beta-amyloid association alters thrombosis and fibrinolysis: a possible contributing factor to Alzheimer’s disease. Neuron..

[65] Lin B, Hasegawa Y, Takane K, Koibuchi N, Cao C, Kim-Mitsuyama S (2016). High-fat-diet intake enhances cerebral amyloid Angiopathy and cognitive impairment in a Mouse model of Alzheimer’s disease, independently of metabolic disorders. J Am Heart Assoc..

[66] Sagare A, Deane R, Bell RD, Johnson B, Hamm K, Pendu R (2007). Clearance of amyloid-beta by circulating lipoprotein receptors. Nat Med.

[67] Drell W, Welch AD (1989). Azarbine-homocystinemia-thrombosis in historical perspective. Pharmacol Ther.

[68] Koldamova R, Schug J, Lefterova M, Cronican AA, Fitz NF, Davenport FA (2014). Genome-wide approaches reveal EGR1-controlled regulatory networks associated with neurodegeneration. Neurobiol Dis.

[69] Sweeney MD, Kisler K, Montagne A, Toga AW, Zlokovic BV (2018). The role of brain vasculature in neurodegenerative disorders. Nat Neurosci.

[70] Barisano G, Montagne A, Kisler K, Schneider JA, Wardlaw JM, Zlokovic BV (2022). Blood–brain barrier link to human cognitive impairment and Alzheimer’s disease. Nat Cardiovasc Res.

[71] Xu S, Edman M, Kothawala MS, Sun G, Chiang L, Mircheff A (2011). A Rab11a-enriched subapical membrane compartment regulates a cytoskeleton-dependent transcytotic pathway in secretory epithelial cells of the lacrimal gland. J Cell Sci.

[72] Lapierre LA, Avant KM, Caldwell CM, Oztan A, Apodaca G, Knowles BC (2012). Phosphorylation of Rab11-FIP2 regulates polarity in MDCK cells. Mol Biol Cell.

[73] Yui N, Lu HAJ, Chen Y, Nomura N, Bouley R, Brown D (2013). Basolateral targeting and microtubule-dependent transcytosis of the aquaporin-2 water channel. Am J Physiol, Cell Physiol.

[74] Takahashi S, Kubo K, Waguri S, Yabashi A, Shin H-W, Katoh Y (2012). Rab11 regulates exocytosis of recycling vesicles at the plasma membrane. J Cell Sci.

[75] Li Q, Xie LH, Haeberle A, Zhang J, Weina P (2006). The evaluation of radiolabeled artesunate on tissue distribution in rats and protein binding in humans. Am J Trop Med Hyg.

[76] Hampel H, Hardy J, Blennow K, Chen C, Perry G, Kim SH (2021). The amyloid-β pathway in Alzheimer’s disease. Mol Psychiatry.

[77] Griffin EF, Yan X, Caldwell KA, Caldwell GA (2018). Distinct functional roles of Vps41-mediated neuroprotection in Alzheimer’s and Parkinson’s disease models of neurodegeneration. Hum Mol Genet.

[78] Kanatsu K, Hori Y, Takatori S, Watanabe T, Iwatsubo T, Tomita T (2016). Partial loss of CALM function reduces Aβ42 production and amyloid deposition in vivo. Hum Mol Genet.

[79] Sadleir KR, Popovic J, Vassar R (2018). ER stress is not elevated in the 5XFAD mouse model of Alzheimer’s disease. J Biol Chem.

[80] Ho WE, Peh HY, Chan TK, Wong WSF (2014). Artemisinins: pharmacological actions beyond anti-malarial. Pharmacol Ther.

[81] Zyad A, Tilaoui M, Jaafari A, Oukerrou MA, Mouse HA (2018). More insights into the pharmacological effects of artemisinin. Phytother Res.

[82] Khan AI, Kapoor A, Chen J, Martin L, Rogazzo M, Mercier T (2018). The antimalarial drug Artesunate attenuates cardiac injury in a rodent model of myocardial infarction. Shock..

[83] Zuo S, Ge H, Li Q, Zhang X, Hu R, Hu S (2017). Artesunate protected blood-brain barrier via sphingosine 1 phosphate receptor 1/phosphatidylinositol 3 kinase pathway after subarachnoid hemorrhage in rats. Mol Neurobiol.

[84] Liu Q-F, Yu H-W, Sun L-L, You L, Tao G-Z, Qu B-Z (2015). Apelin-13 upregulates Egr-1 expression in rat vascular smooth muscle cells through the PI3K/Akt and PKC signaling pathways. Biochem Biophys Res Commun.

[85] Dickerson F, Stallings C, Vaughan C, Origoni A, Goga J, Khushalani S (2011). Artemisinin reduces the level of antibodies to gliadin in schizophrenia. Schizophr Res.

