# Correction: Anti-malaria drug artesunate prevents development of amyloid-β pathology in mice by upregulating PICALM at the blood-brain barrier

**DOI:** 10.1186/s13024-023-00606-7

**Published:** 2023-02-24

**Authors:** Kassandra Kisler, Abhay P. Sagare, Divna Lazic, Sam Bazzi, Erica Lawson, Ching-Ju Hsu, Yaoming Wang, Anita Ramanathan, Amy R. Nelson, Zhen Zhao, Berislav V. Zlokovic

**Affiliations:** Department of Physiology and Neuroscience and the Zilkha Neurogenetic Institute, Keck School of Medicine of the University of Southern California, 1501 San Pablo St, Los Angeles, CA 90089 USA

**Correction: Mol Neurodegener 18, 7 (2023)**

**https://doi.org/10.1186/s13024-023-00597-5**

The original article [1] contained an obsolete version of Additional File 5: Fig. S5 which has since been amended.
