# Supplementary figures and images for: Anti-malaria drug artesunate prevents development of amyloid-β pathology in mice by upregulating PICALM at the blood-brain barrier
Source: Mol Neurodegener. 2023 Jan 27;18:7. doi: 10.1186/s13024-023-00597-5 (PMC9883925; doi:10.1186/s13024-023-00597-5)

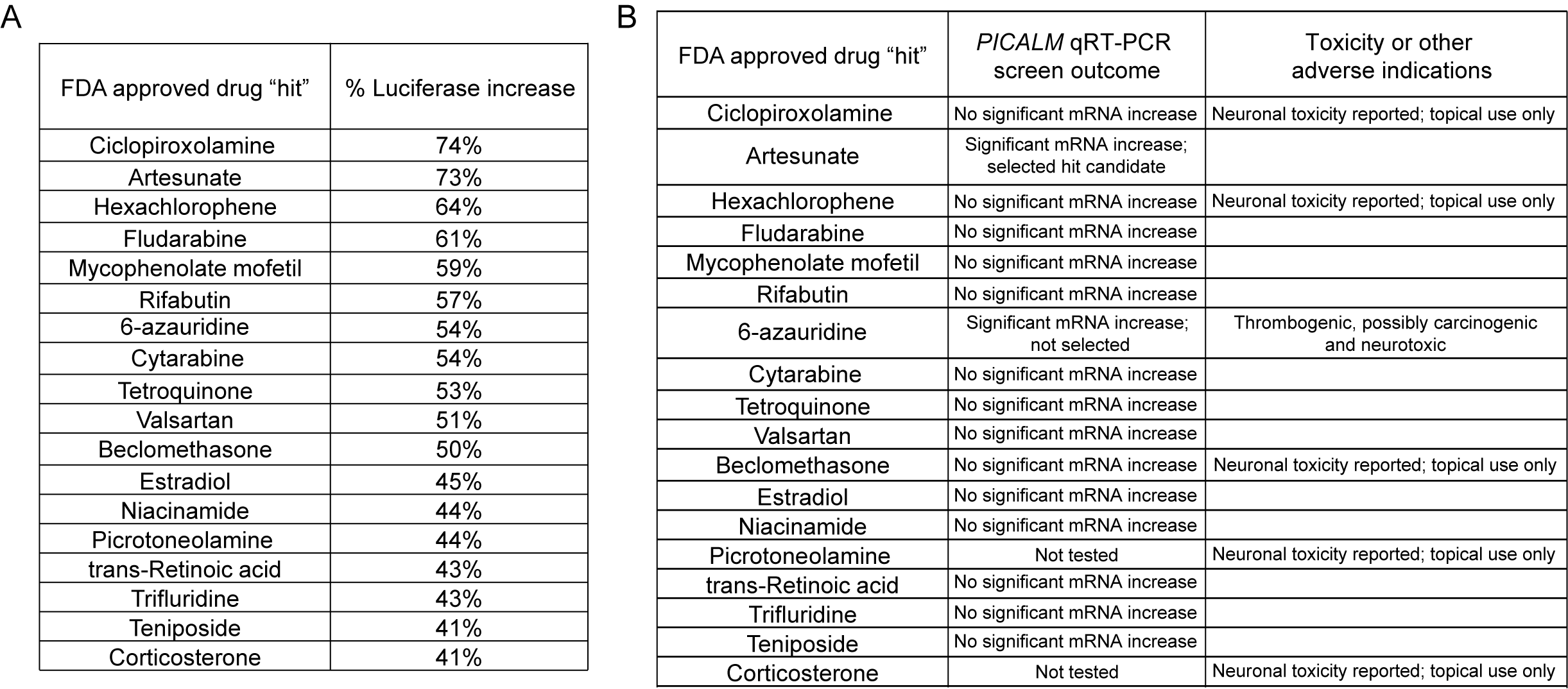

Supplement: Supplementary file 1 — Additional file 1: Fig. S1. Drug hits identified with the HEK293t luciferase reporter assay and a secondary RT-qPCR PICALM mRNA screen in Eahy926 endothelial cells. (A) Unique hits identified by the PICALM; Luciferase screen in HEK293t cells shown in Fig. 1B. Percent increase of luciferase luminescence from screen, normalized to SEAP and DMSO control (See Fig. 1A, B and Methods) are indicated. (B) Table summarizing results of RT-qPCR PICALM mRNA screen in Eahy926 endothelial cells shown in Fig. 1C, and description of toxic or adverse indications reported in literature. Picrotoneoamine and corticosterone were not evaluated further because of their close similarity to other drug hits that did not yield significant PICALM mRNA increases and their reported toxicity. [file 13024_2023_597_MOESM1_ESM.png]

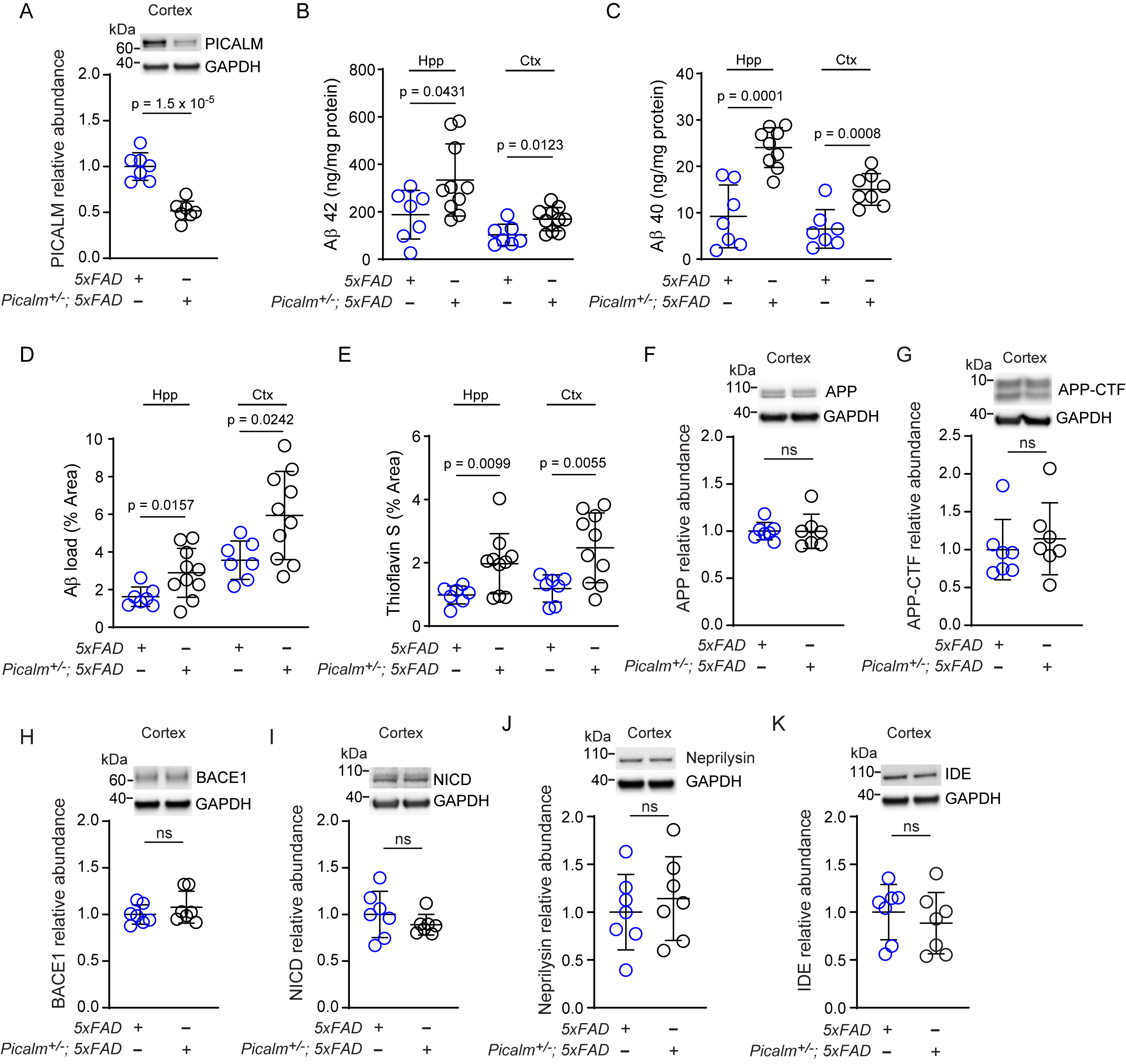

Supplement: Supplementary file 2 — Additional file 2: Fig. S2. Characterization of PICALM loss and amyloid pathology in Picalm+/−; 5XFAD mice compared to 5XFAD littermates. (A) PICALM protein relative expression levels in cortex isolated from 5XFAD mice or littermate Picalm+/−; 5XFAD mice treated with vehicle as in Fig. 2A. (B, C) Amyloid-β 42 (Aβ42) (B) and Aβ40 (C) levels in the hippocampus (Hpp) and cortex (Ctx) in 5XFAD mice or littermate Picalm+/−; 5XFAD mice treated with vehicle. Picalm+/−; 5XFAD data is replotted from Fig. 2C, D. (D) Quantification of Aβ load from pan-Aβ immunostaining in the Hpp and Ctx in 5XFAD mice or littermate Picalm+/−; 5XFAD mice treated with vehicle. Picalm+/−; 5XFAD data is replotted from Fig. 2F. (E) Quantification of Thioflavin S amyloid plaque load in the Hpp and Ctx in 5XFAD mice or Picalm+/−; 5XFAD mice treated with vehicle. Picalm+/−; 5XFAD data is replotted from Fig. 2H. (F-K) Western immunoblotting of Aβ processing proteins and Aβ clearance enzymes. (F) amyloid precursor protein (APP) abundance in cortex, (G) APP C-terminal fragment (APP-CTF) abundance in cortex. (H) β-secretase (BACE1) abundance in cortex. (I) γ–secretase activity as determined by the production of Notch intracellular domain (NICD) fragment from Notch protein, indicated by NICD abundance in cortex, (J) neprilysin abundance in cortex, and (K) insulin degrading enzyme (IDE) abundance in cortex of 5XFAD mice or littermate Picalm+/−; 5XFAD mice treated with vehicle. The relative abundance of proteins was normalized by the house-keeping gene glyceraldehyde 3-phosphate dehydrogenase (GAPDH) protein. Data is single points per mouse indicated by circles, with mean ± SD; n = 7 mice per condition. Significance determined by Student’s two-tail t-test. ns = non-significant by two-tailed t-test. Full blots for A, F-K shown in supp. Fig. 5. [file 13024_2023_597_MOESM2_ESM.png]

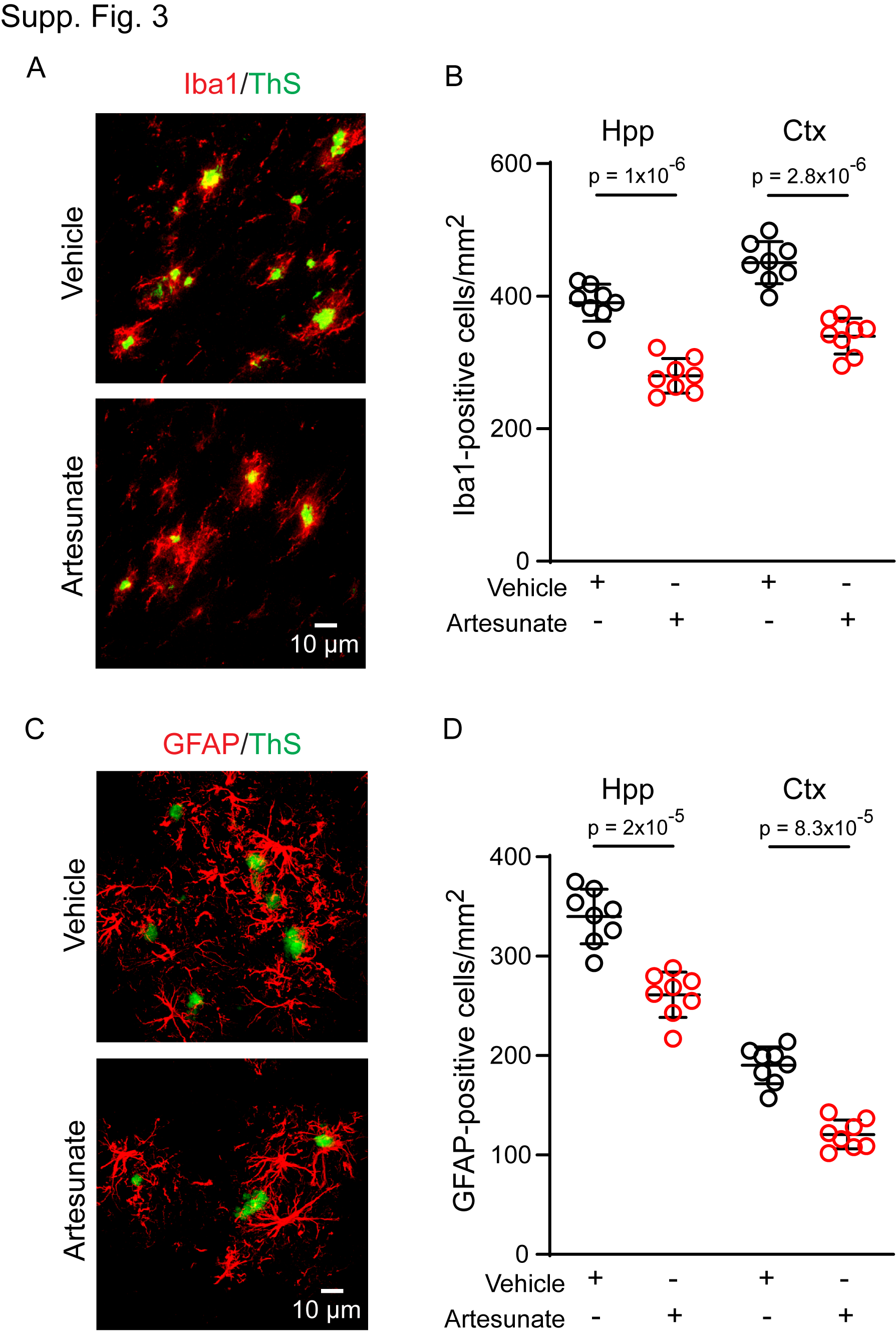

Supplement: Supplementary file 3 — Additional file 3: Fig. S3. Artesunate alleviates microglia and astrocyte responses in Picalm+/−; 5XFAD mice. (A,B) Representative images of Iba1-positive microglia (red) and Thioflavin S-positive amyloid deposits (ThS, green) in cortex (A), and quantification of Iba1-positive microglia in the hippocampus (Hpp) and cortex (Ctx) (B) in Picalm+/−; 5XFAD mice treated with vehicle or artesunate (Art) as shown in Fig. 2A. (C,D) Representative images of GFAP-positive astrocytes (red) and Thioflavin S-positive amyloid deposits (green) in cortex (C), and quantification of GFAP-positive astrocytes in the Hpp and Ctx (D) in Picalm+/−; 5XFAD mice treated with vehicle or Art as shown in Fig. 2A. Scale bars in A, C are 10 μm. n = 8 mice per condition. Single points per mouse indicated by circles in B, D, with mean ± SD. Significance determined by Student’s two-tail t-test in panels B, D. [file 13024_2023_597_MOESM3_ESM.png]

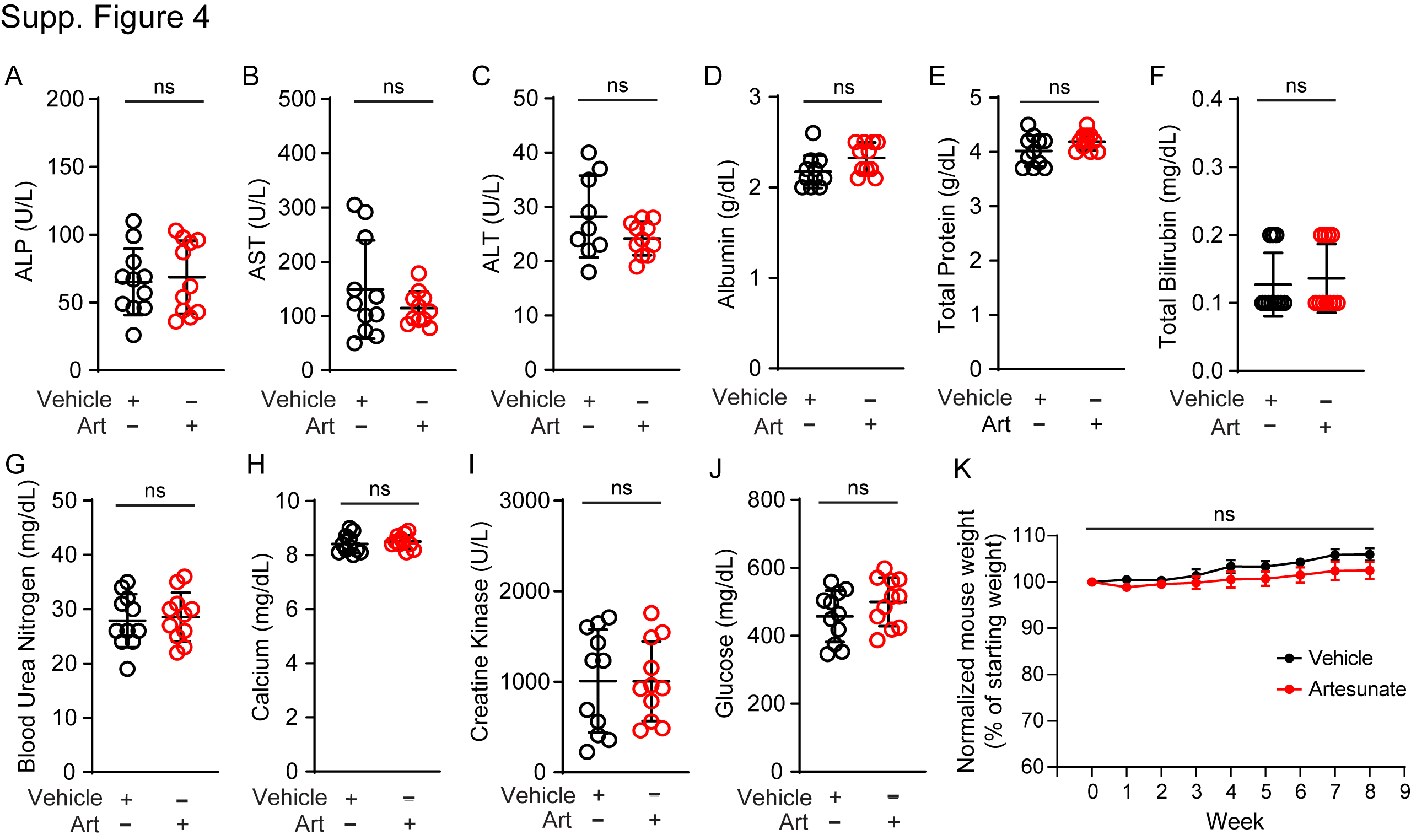

Supplement: Supplementary file 4 — Additional file 4: Fig. S4. Biochemical parameters in blood show no differences between artesunate- and vehicle-treated Picalm+/−; 5XFAD mice. Picalm+/−; 5XFAD mice were treated with vehicle or artesunate (Art) as in Fig. 2A. (A) Alkaline phosphatase (ALP), (B) aspartate aminotransferase (AST), (C) alanine aminotransferase (ALT), (D) albumin, (E) total protein, (F) total bilirubin, (G) blood urea nitrogen, (H) calcium, (I) creatine kinase, and (J) glucose. (K) Mouse weights normalized to treatment starting weight for Picalm+/−; 5XFAD mice treated for two months with vehicle or Art starting at 3 mo of age as in Fig. 2A. A,B, D-J, n = 11 mice per condition; C, n = 9 vehicle and 11 Art treated mice; K, n = 14 vehicle and 15 Art treated mice. A-J, data is single points per mouse indicated by circles in with mean ± SD. In K, data is mean ± SEM. A-E, G-J, statistical significance was determined by Student’s t-test, F by Mann-Whitney U test, and K by two-way ANOVA followed by Bonferroni post-test. ns = non-significant. [file 13024_2023_597_MOESM4_ESM.png]

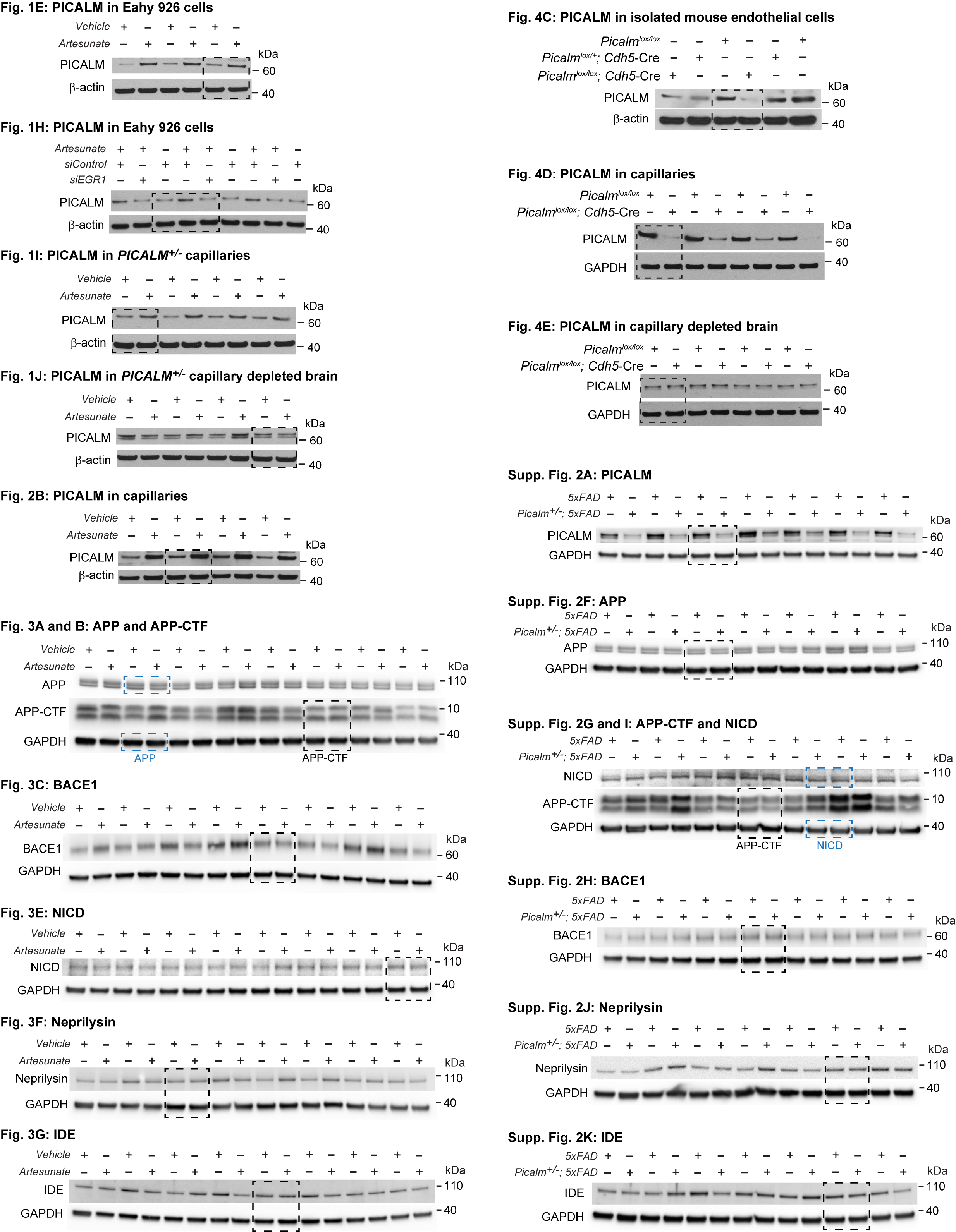

Supplement: Supplementary file 5 — Additional file 5: Fig. S5. Full Western immunoblots for data shown in Figs. 1, 2, 3 and 4 and Supp. Figure 2. Blots for each figure are indicated. Dashed lines indicate bands used as representative images. Blots for Figs. 1, 2, and 4 were imaged with CL-XPosure film, using loading amounts and exposures within the linear dynamic range of the film. Blots for Fig. 3 and S2 were imaged with a Carestream IS4000MM Pro Image Station digital chemiluminescence gel detection instrument within the linear dynamic range of the instrument (see Methods for blotting and detection details). [file 13024_2023_597_MOESM5_ESM.png]
